# Molecular Epidemiology, Evolution and Reemergence of Chikungunya Virus in South Asia

**DOI:** 10.3389/fmicb.2021.689979

**Published:** 2021-06-07

**Authors:** Nadim Sharif, Mithun Kumar Sarkar, Rabeya Nahar Ferdous, Shamsun Nahar Ahmed, Md. Baki Billah, Ali Azam Talukder, Ming Zhang, Shuvra Kanti Dey

**Affiliations:** ^1^Department of Microbiology, Jahangirnagar University, Savar, Bangladesh; ^2^Department of Microbiology, Bangladesh University of Health Sciences, Dhaka, Bangladesh; ^3^Department of Zoology, Jahangirnagar University, Savar, Bangladesh; ^4^Department of Epidemiology and Biostatistics, College of Public Health, University of Georgia, Athens, GA, United States

**Keywords:** chikungunya virus, evolution, epidemiology, phylogeny, South Asia

## Abstract

Chikungunya virus (CHIKV) is a vector (mosquito)-transmitted alphavirus (family *Togaviridae*). CHIKV can cause fever and febrile illness associated with severe arthralgia and rash. Genotypic and phylogenetic analysis are important to understand the spread of CHIKV during epidemics and the diversity of circulating strains for the prediction of effective control measures. Molecular epidemiologic analysis of CHIKV is necessary to understand the complex interaction of vectors, hosts and environment that influences the genotypic evolution of epidemic strains. In this study, different works published during 1950s to 2020 concerning CHIKV evolution, epidemiology, vectors, phylogeny, and clinical outcomes were analyzed. Outbreaks of CHIKV have been reported from Bangladesh, Bhutan, India, Pakistan, Sri Lanka, Nepal, and Maldives in South Asia during 2007–2020. Three lineages- Asian, East/Central/South African (ECSA), and Indian Ocean Lineage (IOL) are circulating in South Asia. Lineage, ECSA and IOL became predominant over Asian lineage in South Asian countries during 2011–2020 epidemics. Further, the mutant E1-A226V is circulating in abundance with *Aedes albopictus* in India, Bangladesh, Nepal, and Bhutan. CHIKV is underestimated as clinical symptoms of CHIKV infection merges with the symptoms of dengue fever in South Asia. Failure to inhibit vector mediated transmission and predict epidemics of CHIKV increase the risk of larger global epidemics in future. To understand geographical spread of CHIKV, most of the studies focused on CHIKV outbreak, biology, pathogenesis, infection, transmission, and treatment. This updated study will reveal the collective epidemiology, evolution and phylogenies of CHIKV, supporting the necessity to investigate the circulating strains and vectors in South Asia.

## Introduction

Chikungunya virus (CHIKV) (family *Togaviridae*) is a mosquito-borne (arthropod-borne virus) *Alphavirus* that causes chikungunya fever in humans (International Committee on Taxonomy of Viruses (ICTV), 2021). Chikungunya virus was first characterized during 1952 to 1953 from a dengue-like outbreak in Newala district, Tanzania. CHIKV is considered as a member of *Semliki Forest* virus (SFV) antigenic group and the word “chikungunya” is derived from Makonde word, Bantu language and translated as “bent over in pain,” describing the posture of CHIKV infected patients (Robinson, 1955; Casals and Whitman, 1957; Spence and Thomas, 1959; Hammon et al., 1960; Jupp et al., 1988; Ekstrom et al., 1994; Gubler, 2001; Weaver et al., 2005; Weaver and Lecuit, 2015). About 3–5 million cases of CHIKV are reported every year globally (World Health and Organization (WHO), 2021). Although mortality due to CHIKV infection is rare, infection of CHIKV can cause severe and long term health conditions in patients (Deller and Russell, 1968). CHIKV can cause both symptomatic and asymptomatic infections in humans (Deller and Russell, 1968; Silva et al., 2018). Symptomatic CHIKV infection is categorized into acute, chronic and atypical depending on the manifestation of symptoms (Fourie and Morrison, 1979; Silva et al., 2018; Vairo et al., 2019). Several clinical symptoms of CHIKV infection overlap with the symptoms of dengue virus (DENV) and Zika virus (ZIKV) infections (Myers and Carey, 1967; Nimmannitya et al., 1969; Carey, 1971; Edwards et al., 2016; Furuya-Kanamori et al., 2016; Gaibani et al., 2016; Rodriguez-Morales et al., 2016; Jain et al., 2018; Ng et al., 2018; Silva et al., 2018; Zanotto and Leite, 2018). The co-circulation of CHIKV with other significant arboviruses, such as dengue virus, Zika virus, mayaro virus (MAYV), and yellow fever virus (YFV) in tropical regions of Asia with overlapping symptoms requires continuous epidemiological surveillance and effective differential diagnosis strategies (Halstead et al., 1969; Weaver and Lecuit, 2015; Vairo et al., 2019).

Chikungunya virus is a small (∼70 nm-diameter), enveloped virus with a linear, positive strand RNA genome of ∼11.8 kilo-bases (Khan et al., 2002; Kawashima et al., 2014). The RNA genome consists of one non-translated region (NTR) at 5′, two ORFs and another non-translated region (NTR) at 3′ end. Two polyproteins are encoded by two major open reading frames (ORFs) in CHIKV (Simizu et al., 1984; Schlesinger and Schlesinger, 1986). The positive-sense 5′ two-third RNA genome directly encodes a polyprotein containing four non-structural proteins (nsP1–4). The structural proteins are encoded by 3′ one-third of the genome (Simizu et al., 1984). The structural polyprotein converts into a capsid protein, two major envelope surface glycoproteins (E1 and E2) as well as two small peptides, E3 and 6K (Simizu et al., 1984).

Transmission of CHIKV involves two major cycles depending on the region of circulation. In African regions, CHIKV circulates mainly in a sylvatic/enzootic cycle involving forest dwelling mosquitoes and non-human primates (NHP) (Silva et al., 2018; Simo et al., 2019). The viruses rely on NHP as reservoir (e.g., monkeys and other vertebrates) hosts during inter-epidemic periods and transmitted by *Aedes* (e.g., *Aedes furcifer* and *Aedes africanus*) mosquitoes from reservoirs to human during epidemic (Jupp et al., 1988; Jupp and McIntosh, 1990; Pialoux et al., 2007; Powers and Logue, 2007; LaBeaud et al., 2011). On the contrary in urban cycles, a mosquito to (and from) human transmission is maintained. The urban cycle of CHIKV has been associated with several large epidemics of CHIKV across different continents including Asia, Europe, and North America (Weaver and Lecuit, 2015; Silva et al., 2018). Two significant species of mosquitoes namely, *Aedes aegypti* and *Aedes albopictus* (the “tiger” mosquito) are mainly involved in urban transmission of the disease (Soekiman, 1987; McGill, 1995; Reiter et al., 2006; Weaver and Lecuit, 2015; Wahid et al., 2017; Silva et al., 2018; Vairo et al., 2019). In temperate climates, *Ae. albopictus* mosquitoes thrive in high density (Soekiman, 1987; Reiter et al., 2006). *Ae. albopictus* are expanding into and adapting to new areas with the potential transmission capability of CHIKV and have been involved in recent epidemics in Asia (Soekiman et al., 1986a, b; Soekiman, 1987; Reiter et al., 2006; Weaver and Lecuit, 2015).

Persistent and larger epidemics of CHIKV infecting millions of people have been reported from Asia, but limited numbers of epidemiological research has been undertaken (Powers and Logue, 2007; Wahid et al., 2017; Vairo et al., 2019). South Asian regions are endemic for CHIKV epidemics. Continuous surveillance including phylogenetic, evolutionary, and epidemiologic analyses are required in these endemic regions to catch up the contemporary changes in the virus for developing effective diagnostics, treatments, and vaccines (Myers and Carey, 1967; Edwards et al., 2016; Rodriguez-Morales et al., 2016; Ng et al., 2018). In this review, updated epidemiology, evolution and phylogenomics of CHIKV in South Asia during 2004–2020 have been evaluated. Clinical features of chikungunya fever, transmission in temperate and tropical regions and the laboratory testing for the disease are also described in this study. Particularly, this study focuses on the recent trends of CHIKV epidemic in South Asia to create an integrated baseline for future studies.

## Molecular Epidemiology, Phylogeny and Evolution of Chikungunya Virus in South Asia and Rest of the World

Starting from Africa, CHIKV has been transmitted globally. Recently, CHIKV infection has been detected from different countries on all continents, except Antarctica. Retrospective case studies have suggested that CHIKV epidemics have occurred during 1760s (Ross, 1956; Jupp et al., 1988). During early epidemics, they were inaccurately documented as dengue virus infection (Brighton et al., 1983; Silva and Dermody, 2017). CHIKV was first isolated and characterized from the serum of an infected patient with dengue like symptoms in Tanzania during 1952 to 1953 (Casals and Whitman, 1957; Johnson et al., 1977). CHIKV was detected in South Asia in a short time after identification in East Africa (1952) (Ross, 1956; Powers and Logue, 2007; Silva and Dermody, 2017; Wahid et al., 2017; Simo et al., 2019). After the first identification, local and occasional outbreaks of CHIKV were recorded for the following ∼50 years before 2004 in many countries in Asia (Mason and Haddow, 1957; Peyrefitte et al., 2007; Silva and Dermody, 2017; Simo et al., 2019).

**FIGURE 1:**
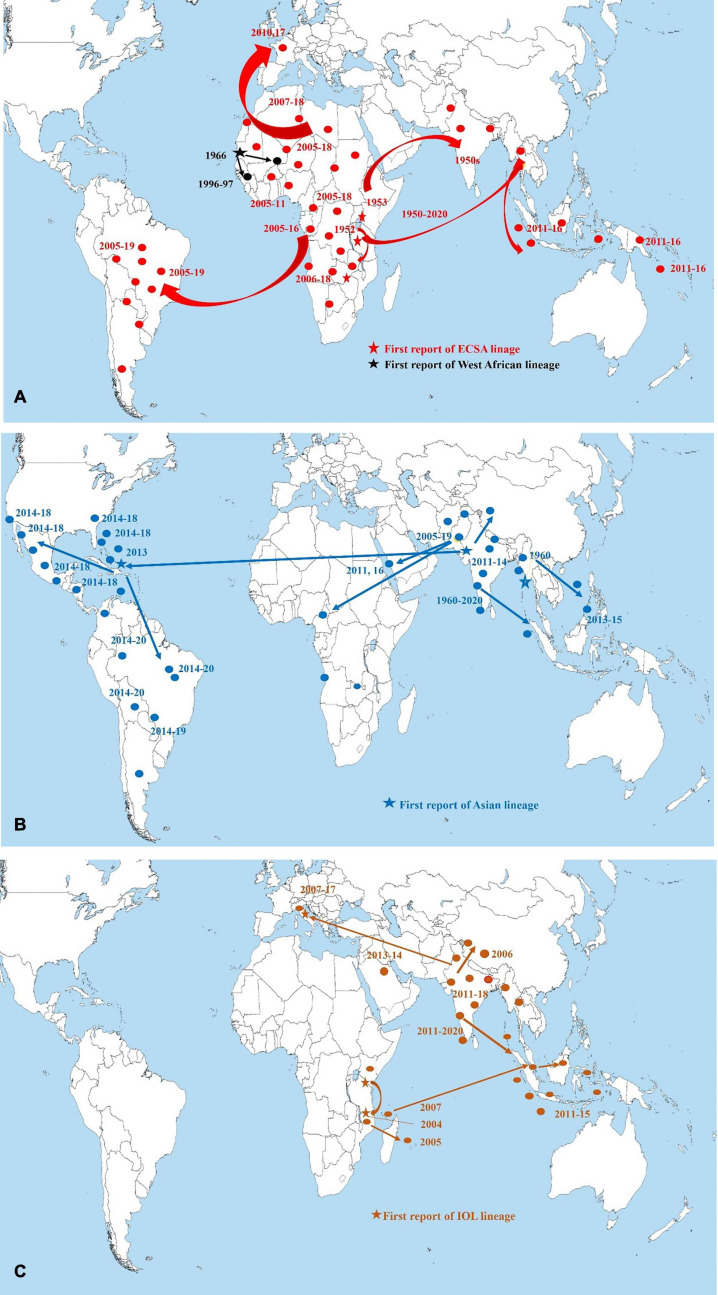
Map depicting worldwide distribution of chikungunya virus lineages during 1950s to 2020. Star signs indicate the first report/start of large outbreak of chikungunya virus. Circles indicate local outbreaks. **(A)** Red circles, arrows, and stars indicate ECSA lineage, black circles, arrows, and stars indicate West African lineage. **(B)** Navy blue circles, arrows, and stars indicate Asian lineage. **(C)** Brown circles, arrows, and stars indicate IOL lineage (Centers for Disease Control and Prevention (CDC), 2021; European Centre for Disease Prevention and Control (ECDC), 2021; Pan American and Health Organization (PAHO), 2021; World Health and Organization (WHO), 2021). The arrows indicated the probable spread of CHIKV based on the information extracted from Centers for Disease Control and Prevention (CDC), 2021; European Centre for Disease Prevention and Control (ECDC), 2021; Pan American and Health Organization (PAHO), 2021; World Health and Organization (WHO), 2021. Data were extracted directly from the databases and websites and cross-checked. The map was built using ArcGIS Pro software.

**FIGURE 2:**
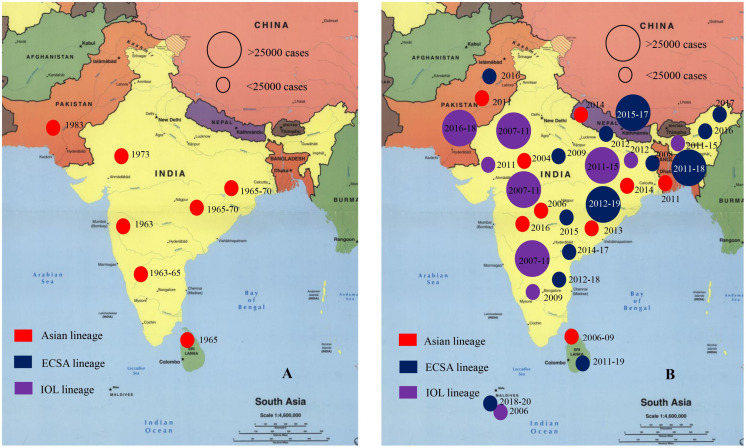
Onset of chikungunya virus outbreak in South Asia regions. **(A)** Indicates the case number of CHIKV patients during 1950s to 2003 in South Asia. **(B)** Indicates the case number of CHIKV patients during 2004 to 2020 in South Asian countries (Centers for Disease Control and Prevention (CDC), 2021; World Health and Organization (WHO), 2021). Star represents capital and large cities.

Chikungunya virus has remained endemic in South Asian countries since 1960s (Powers and Logue, 2007; Wimalasiri-Yapa et al., 2019). The first significant CHIKV outbreak in Asia was reported in the early 1960s in Bangkok, Thailand (Marchette et al., 1978; Powers and Logue, 2007). Outbreaks have been documented from seven countries out of eight in South Asia (Powers et al., 2000; Powers and Logue, 2007; Wimalasiri-Yapa et al., 2019). In SA and SEA, local and minor outbreaks of CHIKV were reported during the 1960s to 1980s in India, Indonesia, Malaysia, Cambodia, Vietnam, Myanmar, Pakistan, and Thailand (Pavri, 1964; Bedekar and Pavri, 1969; Burke et al., 1985; Kit, 2002; Porter et al., 2004; Zim et al., 2013; Weaver and Lecuit, 2015; Silva and Dermody, 2017; Wimalasiri-Yapa et al., 2019). Before 1985, most of the CHIKV outbreaks dated during the period 1961–1970 including several large cities in SEA namely, Kolkata and Bangkok as the main active sites of transmission of CHIKV (Brighton et al., 1983; Powers and Logue, 2007; Wahid et al., 2017). During 1985–2000, no significant outbreaks of CHIKV was documented in South Asian countries (Powers and Logue, 2007; Mascarenhas et al., 2018). Larger outbreaks involving more cases in India, Bangladesh, Pakistan, Sri Lanka, and Maldives have been documented recently (Powers and Logue, 2007; Wimalasiri-Yapa et al., 2019). About 85% of cases of CHIKV had been detected after 2000 in South Asian countries (Padbidri and Gnaneswar, 1979; Powers and Logue, 2007; Hapuarachchi et al., 2010; Manimunda et al., 2010; Haque et al., 2019; Vairo et al., 2019; Wimalasiri-Yapa et al., 2019). After 2007, CHIKV outbreaks in SEA and SA regions were larger and longer that infected millions of people in India, Bangladesh, Bhutan, Nepal, Thailand, and Philippines (Supplementary Table 1; Centers for Disease Control and Prevention (CDC), 2021). In SA, seven countries (Bangladesh, Bhutan, India, Pakistan, Sri Lanka, Nepal, and Maldives) out of eight have reported the local outbreaks and epidemics of CHIKV during 2010 to 2020 (Figures 1A–C, 2; Mallhi et al., 2017; Wahid et al., 2017; Haque et al., 2019; Wimalasiri-Yapa et al., 2019; Centers for Disease Control and Prevention (CDC), 2021).

India has remained as the most affected country with CHIKV (Ravi, 2006; Powers and Logue, 2007; Wimalasiri-Yapa et al., 2019). Increased number of cases of CHIKV were reported after 2007 in India (Saxena et al., 2006; Mavalankar et al., 2007; Seyler et al., 2010; Murhekar et al., 2019). In 2007–2008, outbreak of CHIKV was reported in Bangladesh that continued in 2013, 2014, 2016, and 2017 (Centers for Disease Control and Prevention (CDC), 2021; World Health and Organization (WHO), 2021). In Pakistan, larger outbreaks were reported during 2010–2018 (Powers and Logue, 2007). Further, cases of CHIKV has increased in Maldives, Sri Lanka, Nepal, and Bhutan after 2010 (Supplementary Table 1; Hapuarachchi et al., 2010; Reller et al., 2013; Vairo et al., 2019; Wimalasiri-Yapa et al., 2019). The CHIKV epidemics in South Asia is affected significantly by the ongoing epidemics in other regions in the world. Both the emergence and reemergence of CHIKV in South Asia were linked with outbreaks in Africa. CHIKV was detected and reported repeatedly from various countries in Central and Southern Africa namely, the Central African Republic (CAR), Democratic Republic of Congo (DRC), Malawi, Sudan, Uganda, Zimbabwe, Kenya, and South Africa during the 1960s to the 1990s (Moore et al., 1974; Thonnon et al., 1999; Powers and Logue, 2007; Weaver and Lecuit, 2015; Silva and Dermody, 2017; Wahid et al., 2017; Silva et al., 2018; Simo et al., 2019). After 2005, the outbreaks in Africa significantly contributed in reemergence of CHIKV in South Asia. Reemergence of CHIKV occurred in India in 2007, 2009, 2011, 2015, 2017, and 2019, in Bangladesh during 2009–2011, 2013–2015, 2016, 2017, and 2019, and in Pakistan during 2009, 2011, 2013–2016 (Mallhi et al., 2017; Wahid et al., 2017; Haque et al., 2019; Wimalasiri-Yapa et al., 2019; Centers for Disease Control and Prevention (CDC), 2021).

As of 2020, local and autochthonous outbreaks have been reported from about 114 countries and territories (Centers for Disease Control and Prevention (CDC), 2021). The first larger outbreak occurred in coastal Kenya in 2004 (Powers and Logue, 2007; Sergon et al., 2007; Gudo et al., 2016; Silva and Dermody, 2017; Simo et al., 2019). In 2004, two large outbreaks started in Kenya (Powers and Logue, 2007; Weaver and Forrester, 2015; Vairo et al., 2019). This outbreak in Kenya transmitted to the Union of the Comoros by January 2005 (Pastorino et al., 2004). From there CHIKV was transmitted to the surrounding locations in the Indian Ocean. During 2005–2007, about 500,000 cases (1/3 of the population) were documented on La Reunion Island (Josseran et al., 2006; Powers and Logue, 2007; Renault et al., 2007; Mascarenhas et al., 2018). From Indian Ocean, the CHIKV epidemic spread to India, infecting ∼1.5 million individual. Further, the virus was transmitted to Indonesia, Maldives, Sri Lanka, Myanmar, Thailand, and other countries in Asia (Laras et al., 2005; Parola et al., 2006; Silva and Dermody, 2017). First autochthonous transmission in the Americas occurred in 2013 (Yactayo et al., 2016; Humphrey et al., 2017; Mascarenhas et al., 2018). As of 2020, millions of cases of CHIKV have been reported from Latin American countries including Brazil, Bolivia, Colombia, Argentina, Cuba, Costa Rica, Ecuador, and Peru (Powers and Logue, 2007; Rodrigues Faria et al., 2016; Yactayo et al., 2016; Cunha et al., 2020; Centers for Disease Control and Prevention (CDC), 2021; Pan American and Health Organization (PAHO), 2021; World Health and Organization (WHO), 2021). Further, CHIKV infection has transmitted to the Oceania/Pacific islands including the Marshall Islands, American Samoa, Cook Islands, Samoa, French Polynesia, and Kiribati in 2014 (Figures 1A–C; Powers and Logue, 2007; Staples et al., 2009; Vairo et al., 2019; Centers for Disease Control and Prevention (CDC), 2021).

According to previous lineage systems, four lineages namely, East-, Central- and South African lineage (ECSA), Asian Urban lineage (AUL), West African lineage (WA), and Indian Ocean lineage (IOL) were defined (Powers and Logue, 2007; Silva and Dermody, 2017). Recently, the most updated classification system based on 1,066 genomes sampled between February 1953 and December 2019 included nine lineages namely, Asian urban (AUL), AUL-America (AUL-Am), South America (SAL), Middle Africa (MAL), Indian Ocean (IOL), East Africa (EAL), Africa and Asia (AAL), Sister Taxa to ECSA (sECSA) and West Africa (WA) and named according to the regions of origin (de Bernardi Schneider et al., 2019; Nextstrain, 2021; Spicher et al., 2021). Further, genotypes of CHIKV was specified based on partial or complete E1 gene sequencing (Presti et al., 2016; Phadungsombat et al., 2020). Molecular epidemiologic and evolutionary analysis confirmed circulation of several lineages of CHIKV in South Asia (Powers and Logue, 2007; Presti et al., 2016).

East-, Central- and South African lineage is considered as the ancestor and has circulated in South Asia from Africa at early 1960s. The second confirmed lineage, AUL, was first detected in outbreaks in Asian countries (Thailand, India, Cambodia, Vietnam, Malaysia, Taiwan, Myanmar, and Indonesia) during 1958 to 1973 and named as Asian lineage (Powers and Logue, 2007; Presti et al., 2016). Another distinct lineage called Indian Ocean Lineage (IOL) evolved from ECSA lineage (Schuffenecker et al., 2006; Presti et al., 2016; Silva and Dermody, 2017; Pyke et al., 2018) was detected after 2004. Phylogenetic and mutational analysis revealed the presence of Asian lineage, IOL and Africa and Asia lineages in South Asian countries in recent time (Wahid et al., 2017; Deeba et al., 2020). Asian lineage was the most predominant during 1960s to 2000 in South Asian and South East Asian countries (Yadav et al., 2003; Powers and Logue, 2007; Wimalasiri-Yapa et al., 2019). After 2004, the IOL transmitted rapidly in the South Asian countries. After 2005 outbreaks, IOL lineage has been reported from most of the outbreaks (80%) in South Asian countries (Powers and Logue, 2007; Wimalasiri-Yapa et al., 2019). Since 2005, outbreak associated with IOL lineage have emerged and reemerged every year in South Asian countries (Wimalasiri-Yapa et al., 2019; Phadungsombat et al., 2020). Besides, ESCA, AUL, and AAL lineages are circulating in South Asia in a low frequency after 2005 outbreaks. Evolutionary analysis has revealed that during 2010 to 2020, outbreaks in Bangladesh, Bhutan, India, Pakistan, and Sri Lanka have been associated with ECSA-IOL lineage (Powers and Logue, 2007; Wahid et al., 2017; Melan et al., 2018; Deeba et al., 2020). During 2017 outbreaks in Bangladesh, only the ECSA-IOL lineage was reported (Supplementary Table 1). Other countries of South East Asia, namely, Bhutan, Myanmar, and Vietnam had reported the presence of IOL lineage during recent outbreaks (Powers and Logue, 2007; Staples et al., 2009; Pyke et al., 2018; Wimalasiri-Yapa et al., 2019).

**FIGURE 3:**
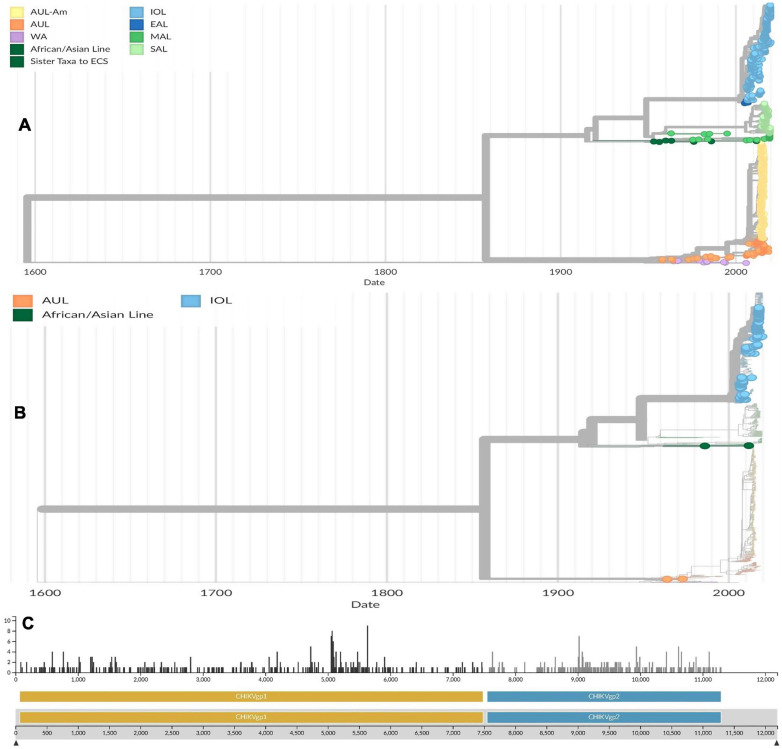
**(A)** Phylogenetic tree of globally distributed 1,066 genomes of chikungunya viruses sampled between February 1953 and December 2019. The phylogenetic tree was retrieved and from Nextstrain and modified based on the most updated information on CHIKV (de Bernardi Schneider et al., 2019; Spicher et al., 2021). **(B)** Phylogenetic tree of 157 whole genomes of CHIKV circulating in South Asia (India-88, Bangladesh-40, Sri Lanka-22, and Pakistan-7) sampled between February 1953 and December 2019. The trees were conducted using Maximum likelihood model. The trees were computed using bootstrap value of 1,000. Reference sequences were selected using temporal and spatial emergence of isolates during outbreaks in South Asia (de Bernardi Schneider et al., 2019; Spicher et al., 2021). **(C)** Mutational analysis of available reference genomes of CHIKV circulating in South Asian countries. The horizontal scale indicated the nucleotide base position in 5′ to 3′ direction, while the vertical scale indicated the number of substitutions per position in the genome (Nextstrain, 2021). Lineages are indicated as Asian urban (AUL), AUL-America (AUL-Am), South America (SAL), Middle Africa (MAL), Indian Ocean (IOL), East Africa (EAL), Africa and Asia (AAL), Sister Taxa to ECSA (sECSA), and West Africa (WA) (Nextstrain, 2021). Source: https://nextstrain.org/community/ViennaRNA/CHIKV.

Molecular evolutionary analysis confirmed the divergence of lineages from each other in previous studies and Nextstrain project and recently published phylogenies and evolutionary analysis (de Bernardi Schneider et al., 2019; Deeba et al., 2020; Nextstrain, 2021; Spicher et al., 2021). The single nucleotide variants of CHIKV can change the stability and fold of locally stable RNA structures. Besides, the 3′ untranslated regions of CHIKV was found to contain non-structural RNA elements and evolutionary conserved regions (de Bernardi Schneider et al., 2019; Deeba et al., 2020; Spicher et al., 2021). Difference among lineages and origin of one lineage from other can be traced by analyzing duplication events and changes of architecture in 3′UTR (de Bernardi Schneider et al., 2019). An estimation of average evolutionary divergence over sequence pairs within CHIKV lineages was calculated in previous studies by following the maximum likelihood model (de Bernardi Schneider et al., 2019). The number of base substitutions per site was expressed from averaging over all sequence pairs within each group and found that AUL was most divergent (substitution per site was 0.0128) followed by MAL (0.0107) and WA (0.0102), while SAL was least divergent (0.003). In four countries, the available 157 whole genome of CHIKV in Nextstrain (India-88, Bangladesh-40, Sri Lanka-22, and Pakistan-7) had an estimated 2.63e^–4^ substitution per site per year (Figures 3A–C). The number of mutations including substitutions are higher in CHIKVgp1 within 5,000 bases to 6,000 bases position, while in CHIKVgp2 the frequency of mutation is about 1.9 per position within 8,500 bases to 11,000 bases.

## Transmission of CHIKV

Chikungunya virus is transmitted in humans by infected mosquitoes (Diallo et al., 1999). CHIKV is an enzootic virus in tropical regions of Africa and Asia (Paul and Singh, 1968; Mourya, 1987; Silva et al., 2018). Emergence and reemergence of CHIKV is significantly regulated by the transmission of the virus through vectors. To understand the reemergence potential in South Asia, this review covered the transmission of CHIKV. Generally, an uninfected mosquito takes in CHIKV from infected viremic person during ingesting the blood (Mourya and Banerjee, 1987; Monteiro et al., 2019). The virus is replicated inside the mosquito midgut. When CHIKV carrying mosquitoes bite a healthy individual, the virus is transmitted inside his/her body (Silva et al., 2018). The virus also replicates inside newly infected person body (Silva and Dermody, 2017). If another uninfected mosquito bite the newly infected person after he has become viremic, the mosquito will take in CHIKV and start another cycle (Silva et al., 2018; Onyango et al., 2020). The complete transmission cycle from human to mosquito and back to humans can be completed within a week (Diallo et al., 1999). Mosquitoes can act as vectors of CHIKV. Both vertical and horizontal transmissions of the virus can occurred in mosquitoes (Mavale et al., 2010; Jain et al., 2016). For successful transmission from arthropod vectors to a human, CHIKV must replicate inside the vectors and reach the salivary glands within 1 week (Lim et al., 2018).

Transmission of CHIKV is maintained by sylvatic cycle in the African and urban cycle in the Asian regions (Jupp and McIntosh, 1990; Staples et al., 2009; Monteiro et al., 2019). In South Asia, the urban mosquito *Ae. aegypti* and *Ae. albopictus* have been reported to be the most significant vector (Soekiman et al., 1986a, b; Banerjee et al., 1988; Mourya et al., 1994; Diallo et al., 1999; Scolari et al., 2019). Regional large outbreaks in South Asia are caused by these urban and peridomestic mosquitoes (Soekiman et al., 1986a, b; Scolari et al., 2019; Wimalasiri-Yapa et al., 2019). *Ae. albopictus* have a great adaption capacity in new ecological niches, as a result it can expand its enzootic range globally (Silva et al., 2018). In urban cycles in South Asia, the onset of epidemics are dependent on environmental factors, viral genetics, mosquito ecology, human behavior, and presence of competent vectors (Soekiman et al., 1986a, b). During the 2005–2006 Indian Ocean Islands epidemic, a substitution point mutation originated at position 226 in the E1 glycoprotein (outer membrane protein) of CHIKV, replacing an Alanine to Valine (Weaver and Lecuit, 2015; Silva et al., 2018). This mutation in ECSA genotype of CHIKV enhanced the vector specificity and epidemic potential of CHIKV (Kumar et al., 2008). The new mutants of CHIKV namely, IOL of ECSA genotype became capable of surviving in and transmitting by *Ae. albopictus* (Tsetsarkin et al., 2016). The E1-A226V substitution increases viral infectivity in *Ae. albopictus* midgut cells without compromising viral replication. This mutant strain initiated autochthonous cases of CHIKV more rapidly through *Ae. albopictus* in South Asian countries (Soekiman et al., 1986a, b; Vazeille et al., 2007; Scolari et al., 2019). Further, *Ae. furcifer–taylori* is the main group of vectors detected during epidemics associated with sylvatic cycle (Jupp and McIntosh, 1990; Scolari et al., 2019). *Ae. furcifer, Aedes taylori, Aedes luteocephalus, Ae. africanus*, and *Aedes neoafricanus* are the major species of vectors involved in sylvatic cycles for many years (Mourya and Banerjee, 1987; Jupp and McIntosh, 1990; Monteiro et al., 2019; Scolari et al., 2019). Numerous field and laboratory works have been undertaken on roles of mosquito vectors in the transmission of CHIKV, but less is known about the importance of vertebrate hosts in viral maintenance (Powers and Logue, 2007; Silva et al., 2018). Laboratory animal studies and serosurveys confirmed the presence of CHIKV specific antibodies in potential vertebrate reservoirs. Significant levels of antibody against CHIKV have been detected in wild non-human primates (Silva et al., 2018).

Vectors and vertebrates have significant roles in inter-epidemic periods both in the sylvatic and urban transmission. In sylvatic cycles, non-human primate (NHP) species including Guinea baboons, Chacma baboons, African green monkeys, patas monkeys, red-tail monkeys, guenons, bushbabies, and mandrills may have significant roles as amplifiers hosts or reservoirs of CHIKV (Silva et al., 2018). On the contrary, in urban cycle, the mosquitoes play main roles probably by *trans-*ovarian (vertical transmission) cycles (Silva et al., 2018).

## Clinical Features of Patients Infected With CHIKV

**FIGURE 4:**
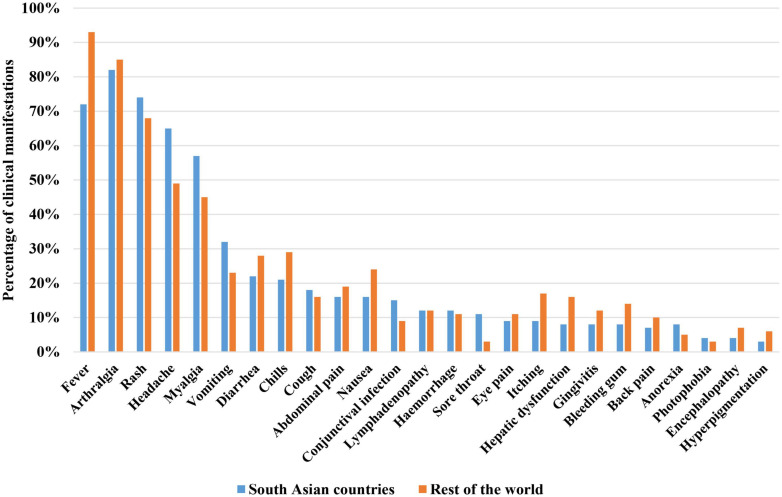
Comparison of clinical manifestations of patients infected with CHIKV in South Asia and rest of the world (Centers for Disease Control and Prevention (CDC), 2021; European Centre for Disease Prevention and Control (ECDC), 2021; Pan American and Health Organization (PAHO), 2021; World Health and Organization (WHO), 2021).

To understand the complete epidemiological prospects of CHIKV burden in South Asian countries, studies on the clinical manifestations in patients infected with CHIKV are required. Generally, the incubation period of CHIKV in human ranges from 3 days to 7 days (Munasinghe et al., 1966; Powers and Logue, 2007; Staples et al., 2009; Onyango et al., 2020). Most of the studies on CHIKV infection clinical presentation reported that about 70–93% of the patients develop symptoms, 3–25% seropositive patients may be asymptomatic, and 2–7% patients may develop atypical symptoms (Powers and Logue, 2007; Staples et al., 2009; Silva et al., 2018; Suhrbier, 2019; Wimalasiri-Yapa et al., 2019). The most reported triad of clinical signs and symptoms for CHIKV infection from documented epidemics and outbreaks includes fever, arthralgia (joint pain), and a rash (itchy rash) (Munasinghe et al., 1966; Riswari et al., 2016). Most of the time the triad is accompanied by other symptoms of the CHIKV infection. Generally, epidemics of CHIKV infection result in two clinical outcomes of illness including the acute phase and chronic phase (Silva et al., 2018; Suhrbier, 2019). In most of the cases, fever accompany with the joint pain and rash. Rash is reported from 50 to 60% cases (Silva and Dermody, 2017). The non-itchy rash becomes visible during 2–5 days of post-infection (Powers and Logue, 2007; Silva et al., 2018). After fever, the most significant clinical presentation of CHIKV infection is the severe joint pain (arthralgia) (Powers and Logue, 2007; Suhrbier, 2019). Arthralgia is reported from about 90 to 98% of CHIKV cases (Brighton and Simson, 1984; Silva and Dermody, 2017). Besides triad, weakness, malaise, headache, chills, retro-orbital pain, photophobia, lumbar back pain, conjunctivitis, pharyngitis, lymphadenopathy and myalgia are other common symptoms reported with CHIKV infection (Table 1; Silva and Dermody, 2017; Silva et al., 2018; Suhrbier, 2019; Wimalasiri-Yapa et al., 2019). Most infections completely resolve within weeks or months but there have been documented cases of CHIKV-induced arthralgia persisting for several years with up to 12% of patients with CHIKV disease developing chronic joint problems (Powers and Logue, 2007; Silva et al., 2018). A comparison of clinical manifestations associated with CHIKV in South Asia and rest of the world is presented in Figure 4.

**TABLE 1:** Distribution of typical and atypical acute clinical manifestations of patients infected with chikungunya virus during 2004 to 2020 in Africa and Asia (Centers for Disease Control and Prevention (CDC), 2021; European Centre for Disease Prevention and Control (ECDC), 2021; Pan American and Health Organization (PAHO), 2021; World Health and Organization (WHO), 2021).

**Organ/System**	**Percentage of patients (*N* = 1022108)**	**Typical acute clinical manifestations**	**Atypical acute clinical manifestations (%)**
Systemic	85–100%	Fever	Lymphadenopathy (5)
		Asthenia	
Musculoskeletal	70–90%	Arthralgia	Articular destruction (7)
		Arthritis	Bleeding gums
		Myalgia	
		Joint edema	
		Tenosynovitis	
		Backache	
		Relapsing-remitting polyarthralgias	
Skin	65–75%	Rash	Bullous dermatosis
		Erythema	Hyperpigmentation
		Bodyache	Stomatitis
			Melena
			Xerosis (10)
Neurological	45–55%	Headache	Meningoencephalitis
		Fatigue	Encephalopathy Seizures
			Sensorineural Abnormalities
			Guillain-Barré syndrome
			Paresis
			Palsies
			Neuropathy (30–40)
Cardiovascular			Myocarditis
			Pericarditis
			Arrhythmias
			Hypotension
			Cardiomyopathy
			Heart failure (20)
Gastrointestinal			Nausea Vomiting
			Abdominal pain
			Anorexia
			Diarrhea (<5)
Hematological	5–10%	Lymphopenia	Hemorrhage (<5)
		Thrombocytopenia	
Respiratory			Dyspnea
			Pneumonia
			Respiratory
			Failure (14–25)
Ocular	5–10%	Retro-orbital pain	Conjunctivitis Photophobia
		Photosensitivity	Retinitis
			Optic neuritis
			Uveitis (<5)
Hepatic			Hepatitis
			Hepatomegaly
			Altered function
			Liver failure (<5)
Renal			Nephritis
			Albuminuria
			Hematuria
			Acute renal failure (20–25)

Long term sequelae associated with CHIKV have been reported in previous studies worldwide (Andrei and De Clercq, 1993; Suhrbier, 2019; Wimalasiri-Yapa et al., 2019). This condition is called CHIKV induced chronic arthralgia (Fourie and Morrison, 1979; Tesh, 1982). Aged persons with prior rheumatological disease are the main victims of chromic symptoms (Tesh, 1982; Adebajo, 1996; Arpino et al., 2009; Barr et al., 2018; Suhrbier, 2019). CHIKV can cause numerous unusual clinical complications in patients (Arpino et al., 2009; Economopoulou et al., 2009; Acevedo et al., 2017; Godaert et al., 2017; Barr et al., 2018). Occasionally, atypical clinical manifestations have been documented from patients infected with CHIKV in South Asia (Suhrbier, 2019; Wimalasiri-Yapa et al., 2019). Atypical symptoms including myocarditis with sinus tachycardia, cardiomegaly, ventricular ectopics, abnormal electrocardiograms and finally congestive heart failure have been reported from Asia (Deller and Russell, 1968; Obeyesekere and Hermon, 1972, 1973; Rajapakse et al., 2010; Silva et al., 2018; Deeba et al., 2019; Wimalasiri-Yapa et al., 2019). In 2015, aggressive clinical course developing shock, severe purpuric lesions, a distinct area large of necrosis in the nasal region, bullous dermatosis, acronecrosis of upper limb, rapid onset of septic shock, and multi-organ failure have been detected in CHIKV infected patients (Powers and Logue, 2007; Rajapakse et al., 2010; Bonifay et al., 2018; Suhrbier, 2019). Vertically infected neonates develop various clinical symptoms (Robillard et al., 2006; Beserra et al., 2019). At the time of birth, about half of the viremic mothers transmit the virus to neonates that results in serious consequences to the neonates (Contopoulos-Ioannidis et al., 2018; van Enter et al., 2018; Shen et al., 2020). About 50% infected neonates develop symptoms within 3–7 days. Numerous clinical symptoms including fever, rashes, sepsis- like illness, poor feeding, diffuse limb edema, irritability (hyperalgesia), respiratory distress, meningoencephalitis, nervous system abnormalities, and hemorrhagic and cardiac manifestations have been detected in neonates (Powers and Logue, 2007; van Enter et al., 2018; Kumar et al., 2019).

## Diagnosis of CHIKV Infection

Diagnosis of CHIKV infection is conducted on the basis of clinical, epidemiological, and laboratory criteria (Silva et al., 2018; Centers for Disease Control and Prevention (CDC), 2021; World Health and Organization (WHO), 2021). Clinical manifestations of CHIKV infection including abrupt onset of high fever and severe joint pain with rash are the main factors of clinical diagnosis. CHIKV infection is difficult to distinguish and diagnose based on only clinical findings in regions where CHIKV co-circulates with DENV and ZIKV (Calisher, 1999; Cabral-Castro et al., 2016). Diagnostic criteria based on epidemiological findings can include the recent (within last 14 days) travel history of the suspects in areas with CHIKV outbreak or endemics (Centers for Disease Control and Prevention (CDC), 2021; World Health and Organization (WHO), 2021). Laboratory diagnosis of CHIKV infection may include virus isolation, virus characterization, viral RNA detection, and serology (Silva et al., 2018; Centers for Disease Control and Prevention (CDC), 2021; World Health and Organization (WHO), 2021). Immunochromatographic assay targeting the E1 antigen of virus from sera of patients can detect different CHIKV genotypes (Silva et al., 2018; Centers for Disease Control and Prevention (CDC), 2021; World Health and Organization (WHO), 2021). Molecular methods including RT-PCR, RT-LAMP, qRT-PCR are most reliable to diagnose CHIKV because of high sensitivity and specificity (Calvo et al., 2016). In endemic regions of DENV, ZIKV, and *Leptospira* infections differential diagnosis by novel multiplex molecular methods have been introduced to detect CHIKV. Among them, RT-LAMP assay is the most promising in differentiating between ZIKV, CHIKV, and DENV infections (Silva et al., 2018; Centers for Disease Control and Prevention (CDC), 2021; World Health and Organization (WHO), 2021). Further, RT-qPCR assays are also used in differential diagnosis among ZIKV, CHIKV, and DENV (Carey, 1971; Thaung et al., 1975; Furuya-Kanamori et al., 2016; Villamil-Gómez et al., 2016). Serological methods, such as ELISA and plaque reduction neutralization testing (PRNT) are performed generally (Ayu et al., 2010). Various commercial serological test kits are available worldwide (Table 2; Thein et al., 1992; Gaibani et al., 2016; Jain et al., 2018). Diagnosis of CHIKV is prioritized during epidemics, but sporadic cases are often neglected. Further, testing of CHIKV is affected by number and severity of sick people or people suspected of CHIKV infection. Serological test kits and molecular diagnosis can be integrated to detect the sporadic and travelers CHIKV infection rapidly and accurately (Silva et al., 2018; Centers for Disease Control and Prevention (CDC), 2021; World Health and Organization (WHO), 2021). In South Asia, the co-circulation of DENV-CHIKV is one of the main concerns in diagnosis approaches. After the rainy season in 2019, a larger outbreak of DENV was reported to infect about 0.2 million people in Bangladesh (Centers for Disease Control and Prevention (CDC), 2021; World Health and Organization (WHO), 2021). During the outbreak of DENV in Bangladesh CHIKV remained under-diagnosed or undiagnosed that misrepresented the actual burden of CHIKV. Appropriate etiological diagnosis should be achieved through combined clinical, epidemiological and laboratory approaches conducted by expert health professionals.

**TABLE 2:** Commercially available Chikungunya Virus (CHIKV) diagnostic assays in South Asia with principle of functions (Gaibani et al., 2016; Centers for Disease Control and Prevention (CDC), 2021; World Health and Organization (WHO), 2021).

**Manufacturer**	**Country of origin**	**Principle**	**IgM**	**IgG**
Abcam	Germany	IgM human ELISA kit	+	/
CTK Biotech	United States	CHIK IgM combo rapid test	+	/
CTK Biotech	United States	RecombiLISA CHIK IgM	+	
DRG*	Germany	CHIK IgM micro-capture ELISA	+	/
DRG	Germany	ELISA	/	+
Euroimmun	Germany	Anti-CHIKV IIFT	+	+
Euroimmun	Germany	Anti-CHIKV ELISA	+	+
GenWay	Germany	IgM-capture ELISA	+	/
GenWay	Germany	ELISA	/	+
IBL international	Germany	IgM micro-capture ELISA	+	/
IBL international	Germany	IgG-capture ELISA	/	+
InBios	United States	CHIKjj-MAC-ELISA	+	+
Novatec	Germany	IgM-capture ELISA	+	/
Novatec	Germany	ELISA	/	+
SD Diagnostics	South Korea	CHIKa IgM ELISA	+	/
SD Diagnostics	South Korea	SD BIOLINE Chikungunya IgM	+	/

*Only for research purposes.

## The Risk of CHIKV Epidemics in South Asia in Future

The previous epidemiological studies support for a larger outbreak of CHIKV in South Asian countries in future. Due to the cyclic nature of CHIKV infection, the epidemics reappear in every 3–4 years in the endemic regions. Further, high density of vectors and co-circulation of CHIKV-DENV during the same seasons are the major concerns in South Asian countries (Thaung et al., 1975; Furuya-Kanamori et al., 2016; Villamil-Gómez et al., 2016). There are various monitoring systems for CHIKV in South Asian countries, but they are only applied during epidemics. As a result, the real disease burden of CHIKV in South Asia still remains underrated. Further, the number of existing genotypic characterization of CHIKV is not enough to point out the diversity in South Asia. The evolutionary and epidemiologic analysis in this article supports for a severe and prolonged epidemics of CHIKV in south Asian countries in near future. To manage such an epidemic in future, this study suggests to conduct routine genotypic surveillance and genomic characterizations of CHIKV to assess the actual diversity of the virus in South Asia. Further, to mitigate the risk of larger epidemic, integrated approaches including epidemiologic characterizations, vector surveillance, evolutionary analysis and effective routine diagnosis are required to reduce the risk of future outbreaks and associated health burden.

## Conclusion

In conclusion, this study finds that CHIKV has become a consistent health burden in South Asia. Tropical regions of South Asia and South America have been the main focal point of CHIKV transmission after 2007. Recently, larger epidemics of CHIKV involving millions of people have been reported from India, Bangladesh, Nepal, Bhutan, and Pakistan. Three lineages of CHIKV namely, Asian, ECSA and IOL are circulating in South Asia during 2011–2020. After 2011, ECSA lineage and IOL lineage of genotype ECSA has become predominant in South Asian countries. Prevalence of E1-A226V mutants and density of vectors namely, *Aedes aegypti* and *Aedes albopictus* remain high in South Asian countries. This study provides a comprehensive analysis on the updated phylogenomic, evolution and molecular epidemiology of CHIKV in South Asian countries, which will not only provide exact scenario of CHIKV but also help in developing better treatment, diagnosis and preventive measures. Further, this study adds integrated knowledge on recent diagnosis, clinical characteristics and transmission of CHIKV in South Asia. The rapid spread of CHIKV in recent years urges the utmost need to take control measures, as well as to search for options to develop vaccines. In future, more studies focusing the molecular characterizations and evolution of CHIKV, as well as vector-pathogen interaction should be conducted to understand the CHIKV infection in depth. This study will work as an updated database for future studies focusing molecular epidemiology, evolution, phylogeny, diagnosis, vaccine development and prevention of CHIKV in South Asia.

## Author Contributions

NS performed the systematic and data collection, provided with the illustrations, and was a major contributor in writing the manuscript. MS performed the data analysis and was a major contributor in revising the manuscript. RF performed the data analysis and was a minor contributor in revising the manuscript. SA performed the data minor analysis. MB performed the minor revision. AT performed the critical evaluation and verification of the manuscript. MZ was a major contributor in revising the manuscript. SD conceptualized the review article and provided oversight, critical evaluation, and verification of the manuscript. All authors read and approved the final manuscript.

## Conflict of Interest

The authors declare that the research was conducted in the absence of any commercial or financial relationships that could be construed as a potential conflict of interest.

## Supplementary Material

Supplementary Table 1 Documented local outbreaks of chikungunya virus with the distribution of vector species during 1950s to 2020 from more than 110 countries and territories worldwide. Data were retrieved from Centers for Disease Control and Prevention (https://www.cdc.gov/chikungunya/index.html), World Health Organization (https://www.who.int/news-room/fact-sheets/detail/chikungunya), European Centre for Disease Prevention and Control (https://www.ecdc.europa.eu/en/chikungunya-monthly), and Pan American Health Organization (https://www.paho.org/hq/index.php?option=com_topics&view= rdmore&cid=5855&Itemid=40931&lang=en).

## References

[B1] Acevedo N. Waggoner J. Rodriguez M. Rivera L. Landivar J. Pinsky B. (2017). Zika virus, chikungunya virus, and dengue virus in cerebrospinal fluid from adults with neurological manifestations. *Front. Microbiol.* 8:42. 10.3389/fmicb.2017.00042 28174559 PMC5258761

[B2] Adebajo A. O. (1996). Rheumatic manifestations of tropical diseases. *Curr. Opin. Rheumatol.* 8 85–89. 10.1097/00002281-199601000-00015 8867545

[B3] Andrei G. De Clercq E. (1993). Molecular approaches for the treatment of hemorrhagic fever virus infections. *Antiviral. Res.* 22 45–75. 10.1016/0166-3542(93)90085-W 8250543

[B4] Arpino C. Curatolo P. Rezza G. (2009). Chikungunya and the nervous system: what we do and do not know. *Rev. Med. Virol.* 19 121–129. 10.1002/rmv.606 19274635

[B5] Ayu S. M. Lai L. R. Chan Y. F. Hatim A. Hairi N. N. Ayob A. (2010). Seroprevalence survey of chikungunya virus in Bagan Panchor. *Am. J. Trop. Med. Hyg.* 83 1245–1248. 10.4269/ajtmh.2010.10-0279 21118929 PMC2990039

[B6] Banerjee K. Mourja D. T. Malunjkar A. S. (1988). Susceptibility & transmissibility of different geographical strains of Aedes aegypti mosquitoes to Chikungunya virus. *Indian J. Med. Res.* 87 134–138. 3397145

[B7] Barr K. L. Khan E. Farooqi J. Q. Imtiaz K. Prakoso D. Malik F. (2018). Evidence of chikungunya virus disease in Pakistan since 2015 with patients demonstrating involvement of the central nervous system. *Front. Public Health.* 6:186. 10.3389/fpubh.2018.00186 30042937 PMC6048291

[B8] Bedekar S. D. Pavri K. M. (1969). Studies with Chikungunya virus. *Indian J. Med. Res.* 57 1193–1197. 5390555

[B9] Beserra F. L. C. N. Oliveira G. M. Marques T. M. A. Farias L. A. B. G. Santos J. R. D. Daher E. D. F. (2019). Clinical and laboratory profiles of children with severe chikungunya infection. *Rev. Soc. Bras. Med. Trop.* 52:e20180232. 10.1590/0037-8682-0232-2018 30994798

[B10] Bonifay T. Prince C. Neyra C. Demar M. Rousset D. Kallel H. (2018). Atypical and severe manifestations of chikungunya virus infection in French Guiana: A hospital-based study. *PLoS One.* 13:e0207406. 10.1371/journal.pone.0207406 30521555 PMC6283639

[B11] Brighton S. W. Prozesky O. W. de la Harpe A. L. (1983). Chikungunya virus infection. A retrospective study of 107 cases. *S. Afr. Med. J* 63 313–315. 6298956

[B12] Brighton S. W. Simson I. W. (1984). A destructive arthropathy following Chikungunya virus arthritis – a possible association. *Clin. Rheumatol.* 3 253–258. 10.1007/BF02030766 6088159

[B13] Burke D. S. Nisalak A. Nimmannitya S. (1985). Disappearance of Chikungunya virus from Bangkok. *Trans. R. Soc. Trop. Med. Hyg.* 79 419–420. 10.1016/0035-9203(85)90398-0 2994265

[B14] Cabral-Castro M. J. Cavalcanti M. G. Peralta R. H. S. Peralta J. M. (2016). Molecular and serological techniques to detect co-circulation of DENV, ZIKV and CHIKV in suspected dengue-like syndrome patients. *J. Clin. Virol.* 82 108–111. 10.1016/j.jcv.2016.07.017 27479173

[B15] Calisher C. H. (1999). “Chikungunya, O’nyong nyong and Mayaro viruses (Togaviridae),” in *Encyclopedia of Virology*, 2nd Edn, eds Granoff A. Webster R. G. (London: Elsevier Science & Technology Books), 236–241.

[B16] Calvo E. P. Sánchez-Quete F. Durán S. Sandoval I. Castellanos J. E. (2016). Easy and inexpensive molecular detection of dengue, chikungunya and zika viruses in febrile patients. *Acta. Trop.* 163 32–37. 10.1016/j.actatropica.2016.07.021 27477452

[B17] Carey D. E. (1971). Chikungunya and dengue: a case of mistaken identity? *J. Hist. Med. Allied Sci.* 26 243–262. 10.1093/jhmas/XXVI.3.243 4938938

[B18] Casals J. Whitman L. (1957). Mayaro virus: a new human disease agent. I. Relationship to other arbor viruses. *Am. J. Trop. Med. Hyg.* 6 1004–1011. 10.4269/ajtmh.1957.6.1004 13487972

[B19] Centers for Disease Control and Prevention (CDC) (2021). *Chikungunya Virus.* Georgia: CDC.

[B20] Contopoulos-Ioannidis D. Newman-Lindsay S. Chow C. LaBeaud A. D. (2018). Mother-to-child transmission of Chikungunya virus: A systematic review and meta-analysis. *PLoS Negl. Trop. Dis.* 12:e0006510. 10.1371/journal.pntd.0006510 29897898 PMC6075784

[B21] Cunha M. S. Costa P. A. Correa I. A. de Souza M. R. Calil P. T. da Silva G. P. D. (2020). Chikungunya Virus: An Emergent Arbovirus to the South American Continent and a Continuous Threat to the World. *Front. Microbiol.* 11:1297. 10.3389/fmicb.2020.01297 32670231 PMC7332961

[B22] de Bernardi Schneider A. Ochsenreiter R. Hostager R. Hofacker I. L. Janies D. Wolfinger M. T. (2019). Updated phylogeny of Chikungunya virus suggests lineage-specific RNA architecture. *Viruses* 11:798. 10.3390/v11090798 31470643 PMC6784101

[B23] Deeba F. Haider M. S. H. Ahmed A. Tazeen A. Faizan M. I. Salam N. (2020). Global transmission and evolutionary dynamics of the Chikungunya virus. *Epidemiol. Infect.* 148:e63. 10.1017/S0950268820000497 32070451 PMC7118414

[B24] Deeba I. M. Hasan M. M. Al Mosabbir A. Siam M. H. B. Islam M. S. Raheem E. (2019). Manifestations of Atypical Symptoms of Chikungunya during the Dhaka Outbreak (2017) in Bangladesh. *Am. J. Trop. Med. Hyg.* 100 1545–1548. 10.4269/ajtmh.19-0122 31038100 PMC6553908

[B25] Deller J. J. Jr. Russell P. K. (1968). Chikungunya disease. *Am. J. Trop. Med. Hyg.* 17 107–111. 10.4269/ajtmh.1968.17.107 5637015

[B26] Diallo M. Thonnon J. Traore-Lamizana M. Fontenille D. (1999). Vectors of chikungunya virus in Senegal: current data and transmission cycles. *Am. J. Trop. Med. Hyg.* 60 281–286. 10.4269/ajtmh.1999.60.281 10072152

[B27] Economopoulou A. Dominguez M. Helynck B. Sissoko D. Wichmann O. Quenel P. (2009). Atypical Chikungunya virus infections: clinical manifestations, mortality and risk factors for severe disease during the 2005–2006 outbreak on Reunion. *Epidemiol. Infect.* 137 534–541. 10.1017/S0950268808001167 18694529

[B28] Edwards T. Signor L. D. C. C. Williams C. Donis E. Cuevas L. E. Adams E. R. (2016). Co-infections with chikungunya and dengue viruses, Guatemala, 2015. *Emerg. Infect. Dis.* 22:2003. 10.3201/eid2211.161017 27767914 PMC5088021

[B29] Ekstrom M. Liljestrom P. Garoff H. (1994). Membrane protein lateral interactions control Semliki Forest virus budding. *EMBO J.* 13 1058–1064. 10.1002/j.1460-2075.1994.tb06354.x 8131740 PMC394913

[B30] European Centre for Disease Prevention and Control (ECDC). (2021). *Chikungunya worldwide overview.* Available online at: https://www.ecdc.europa.eu/en/chikungunya-monthly (accessed Jan 03, 2021).

[B31] Fourie E. D. Morrison J. G. (1979). Rheumatoid arthritic syndrome after chikungunya fever. *S. Afr. Med. J.* 56 130–132. 494034

[B32] Furuya-Kanamori L. Liang S. Milinovich G. Magalhaes R. J. S. Clements A. C. Hu W. (2016). Co-distribution and co-infection of chikungunya and dengue viruses. *BMC Infect. Dis.* 16:84. 10.1186/s12879-016-1417-2 26936191 PMC4776349

[B33] Gaibani P. Landini M. P. Sambri V. (eds) (2016). “Diagnostic methods for CHIKV based on serological tools,” in *Chikungunya Virus*, (New York: Humana Press), 63–73. 10.1007/978-1-4939-3618-2_6 27233261

[B34] Godaert L. Najioullah F. Bartholet S. Colas S. Yactayo S. Cabié A. (2017). Atypical clinical presentations of acute phase chikungunya virus infection in older adults. *J. Am. Geriatr. Soc.* 65 2510–2515. 10.1111/jgs.15004 28940357

[B35] Gubler D. J. (2001). Human arbovirus infections worldwide. *Ann. N. Y. Acad. Sci.* 951 13–24. 10.1111/j.1749-6632.2001.tb02681.x 11797771

[B36] Gudo E. S. Black J. F. Cliff J. L. (2016). Chikungunya in Mozambique: a forgotten history. *PLoS Negl. Trop. Dis.* 10:e0005001. 10.1371/journal.pntd.0005001 27855168 PMC5113865

[B37] Halstead S. B. Nimmannitya S. Margiotta M. R. (1969). Dengue and chikungunya virus infection in man in Thailand, 1962–1964. II. Observations on disease in outpatients. *Am. J. Trop. Med. Hyg.* 18 972–983. 10.4269/ajtmh.1969.18.972 5355243

[B38] Hammon W. M. Rundnick A. Sather G. E. (1960). Viruses associated with epidemic hemorrhagic fevers of the Philippines and Thailand. *Science* 131 1102–1103. 10.1126/science.131.3407.1102 14399343

[B39] Hapuarachchi H. C. Bandara K. B. A. T. Sumanadasa S. D. M. Hapugoda M. D. Lai Y. L. Lee K. S. (2010). Re-emergence of Chikungunya virus in South-east Asia: virological evidence from Sri Lanka and Singapore. *J. Gen. Virol.* 91 1067–1076. 10.1099/vir.0.015743-0 19955565

[B40] Haque F. Rahman M. Banu N. N. Sharif A. R. Jubayer S. Shamsuzzaman A. K. M. (2019). An epidemic of chikungunya in northwestern Bangladesh in 2011. *PLoS One.* 14:e0212218. 10.1371/journal.pone.0212218 30856200 PMC6411100

[B41] Humphrey J. M. Cleton N. B. Reusken C. B. Glesby M. J. Koopmans M. P. Abu-Raddad L. J. (2017). Urban chikungunya in the Middle East and North Africa: a systematic review. *PLoS Negl. Trop. Dis.* 11:e0005707. 10.1371/journal.pntd.0005707 28651007 PMC5501693

[B42] International Committee on Taxonomy of Viruses (ICTV) (2021). *Togaviridae.* Available online at: https://talk.ictvonline.org/ictv-reports/ictv_online_report/positive-sense-rna-viruses/w/togaviridae/872/genus-alphavirus (accessed Jan 03, 2021).

[B43] Jain J. Kushwah R. B. S. Singh S. S. Sharma A. Adak T. Singh O. P. (2016). Evidence for natural vertical transmission of chikungunya viruses in field populations of Aedes Aegypti in Delhi and Haryana states in India-a preliminary report. *Acta Trop.* 162 46–55. 10.1016/j.actatropica.2016.06.004 27282096

[B44] Jain J. Okabayashi T. Kaur N. Nakayama E. Shioda T. Gaind R. (2018). Evaluation of an immunochromatography rapid diagnosis kit for detection of chikungunya virus antigen in India, a dengue-endemic country. *Virol. J.* 15:84. 10.1186/s12985-018-1000-0 29751761 PMC5948817

[B45] Johnson B. K. Chanas A. C. Shockley P. Squires E. J. Gardner P. Wallace C. (1977). Arbovirus isolations from, and serological studies on, wild and domestic vertebrates from Kano Plain, Kenya. Trans. *R. Soc. Trop. Med. Hyg.* 71 512–517. 10.1016/0035-9203(77)90146-8 605465

[B46] Josseran L. Paquet C. Zehgnoun A. Caillere N. Le Tertre A. Solet J. L. (2006). Chikungunya disease outbreak, Reunion Island. *Emerg. Infect. Dis.* 12 1994–1995. 10.3201/eid1212.060710 17354339 PMC3291364

[B47] Jupp P. G. McIntosh B. M. (1990). Aedes furcifer and other mosquitoes as vectors of chikungunya virus at Mica, northeastern Transvaal, South Africa. *J. Am. Mosq. Control Assoc.* 6 415–420. 1977875

[B48] Jupp P. G. McIntosh B. M. Monath T. P. (1988). “Chikungunya virus disease,” in *The Arboviruses: Epidemiology and Ecology. Volume II*, ed. Monath T. P. (Boca Raton, FL: CRC Press), 137–157. 10.1201/9780429280245-7

[B49] Kawashima K. D. Suarez L. A. C. Labayo H. K. M. Liles V. R. Salvoza N. C. Klinzing D. C. (2014). Complete genome sequence of chikungunya virus isolated in the Philippines. *Genome Announc.* 2 e336–e314. 10.1128/genomeA.00336-14 24970822 PMC4073106

[B50] Khan A. H. Morita K. del Carmen, Parquet M. Hasebe F. Mathenge E. G. (2002). Complete nucleotide sequence of chikungunya virus and evidence for an internal polyadenylation siteThe GenBank accession number of the sequence reported in this paper is AF369024. *J. Gen. Virol.* 83 3075–3084. 10.1099/0022-1317-83-12-3075 12466484

[B51] Kit L. S. (2002). Emerging and re-emerging diseases in Malaysia. *Asia. Pac. J. Public Health* 14 6–8. 12597511 10.1177/101053950201400103

[B52] Kumar N. P. Joseph R. Kamaraj T. Jambulingam P. (2008). A226V mutation in virus during the 2007 chikungunya outbreak in Kerala, India. *J. Gen. Virol.* 89 1945–1948. 10.1099/vir.0.83628-0 18632966

[B53] Kumar S. Agrawal G. Wazir S. Kumar A. Dubey S. Balde M. (2019). Experience of perinatal and Neonatal Chikungunya virus (CHIKV) infection in a tertiary care neonatal centre during outbreak in North India in 2016: a case series. *J. Trop. Pediatr.* 65 169–175. 10.1093/tropej/fmy032 29893939

[B54] LaBeaud A. Bashir F. King C. H. (2011). Measuring the burden of arboviral diseases: the spectrum of morbidity and mortality from four prevalent infections. *Popul. Health Metr.* 9:1. 10.1186/1478-7954-9-1 21219615 PMC3024945

[B55] Laras K. Sukri N. C. Larasati R. P. Bangs M. J. Kosim R. Djauzi (2005). Tracking the re-emergence of epidemic chikungunya virus in Indonesia. *Trans. R. Soc. Trop. Med. Hyg.* 99 128–141. 10.1016/j.trstmh.2004.03.013 15693148

[B56] Lim E. X. Y. Lee W. S. Madzokere E. T. Herrero L. J. (2018). Mosquitoes as suitable vectors for alphaviruses. *Viruses* 10:84. 10.3390/v10020084 29443908 PMC5850391

[B57] Mallhi T. H. Khan Y. H. Khan A. H. Tanveer N. Khan O. H. Aftab R. A. (2017). Commentary: outbreak of Chikungunya in Pakistan. *Front. Public Health.* 5:261. 10.3389/fpubh.2017.00261 29034228 PMC5625004

[B58] Manimunda S. P. Sugunan A. P. Rai S. K. Vijayachari P. Shriram A. N. Sharma S. (2010). Outbreak of chikungunya fever, Dakshina Kannada District, South India, 2008. *Am. J. Trop. Med. Hyg.* 83 751–754. 10.4269/ajtmh.2010.09-0433 20889860 PMC2946737

[B59] Marchette N. J. Rudnick A. Garcia R. MacVean D. W. (1978). Alphaviruses in Peninsular Malaysia. I. Virus isolations and animal serology. *Southeast Asian J. Trop. Med. Public Health.* 9 317–329. 34888

[B60] Mascarenhas M. Garasia S. Berthiaume P. Corrin T. Greig J. Ng V. (2018). A scoping review of published literature on chikungunya virus. *PLoS One.* 13:e0207554. 10.1371/journal.pone.0207554 30496207 PMC6264817

[B61] Mason P. J. Haddow A. J. (1957). An epidemic of virus disease in Southern Province, Tanganyika Territory, in 1952–53; an additional note on Chikungunya virus isolations and serum antibodies. *Trans. R. Soc. Trop. Med. Hyg.* 51 238–240. 10.1016/0035-9203(57)90022-6 13443013

[B62] Mavalankar D. Shastri P. Raman P. (2007). Chikungunya epidemic in India: a major public-health disaster. *Lancet Infect. Dis.* 7 306–307. 10.1016/S1473-3099(07)70091-9 17448932

[B63] Mavale M. Parashar D. Sudeep A. Gokhale M. Ghodke Y. Geevarghese G. (2010). Venereal transmission of chikungunya virus by Aedes aegypti mosquitoes (Diptera: culicidae). *Am. J. Trop. Med. Hyg.* 83 1242–1244. 10.4269/ajtmh.2010.09-0577 21118928 PMC2990038

[B64] McGill P. E. (1995). Viral infections: alpha-viral arthropathy. *Baillieres Clin. Rheumatol.* 9 145–150. 10.1016/S0950-3579(05)80151-7 7728877

[B65] Melan A. Aung M. S. Khanam F. Paul S. K. Riaz B. K. Tahmina S. (2018). Molecular characterization of chikungunya virus causing the 2017 outbreak in Dhaka, Bangladesh. *N. Microbes New Infect.* 24 14–16. 10.1016/j.nmni.2018.03.007 29707212 PMC5918166

[B66] Monteiro V. V. S. Navegantes-Lima K. C. de Lemos A. B. Da Silva G. L. de Souza Gomes R. Reis J. F. (2019). Aedes–Chikungunya Virus Interaction: Key Role of Vector Midguts Microbiota and Its Saliva in the Host Infection. *Front. Microbial.* 10:492. 10.3389/fmicb.2019.00492 31024463 PMC6467098

[B67] Moore D. L. Reddy S. Akinkugbe F. M. Lee V. H. David-West T. S. Causey (1974). An epidemic of chikungunya fever at Ibadan, Nigeria, 1969. *Ann. Trop. Med. Parasitol.* 68 59–68. 10.1080/00034983.1974.11686925 4152305

[B68] Mourya D. T. (1987). Absence of transovarial transmission of Chikungunya virus in Aedes aegypti & Ae. albopictus mosquitoes. *Indian J. Med. Res.* 85 593–595. 3666861

[B69] Mourya D. T. Banerjee K. (1987). Experimental transmission of chikungunya virus by Aedes vittatus mosquitoes. *Indian J. Med. Res.* 86 269–271. 3428961

[B70] Mourya D. T. Gokhale M. D. Malunjkar A. S. Bhat H. R. Banerjee K. (1994). Inheritance of oral susceptibility of Aedes aegypti to chikungunya virus. *Am. J. Trop. Med. Hyg.* 51 295–300. 10.4269/ajtmh.1994.51.295 7943547

[B71] Munasinghe D. R. Amarasekera P. J. Fernando C. F. (1966). An epidemic of dengue-like fever in Ceylon (chikungunya) – a clinical and haematological study. *Ceylon Med. J.* 11 129–142. 5984948

[B72] Murhekar M. Kanagasabai K. Shete V. Joshua V. Ravi M. Kirubakaran B. K. (2019). Epidemiology of chikungunya based on laboratory surveillance data—India, 2016–2018. *Trans. R. Soc. Trop. Med. Hyg.* 113 259–262. 10.1093/trstmh/try141 30715511

[B73] Myers R. M. Carey D. E. (1967). Concurrent isolation from patient of two arboviruses, chikungunya and dengue type 2. *Science* 157 1307–1308. 10.1126/science.157.3794.1307 6038994

[B74] Nextstrain (2021). *Molecular epidemiology of Chikungunya virus.* Available online at: https://nextstrain.org/community/ViennaRNA/CHIKV?f_country=Bangladesh,Pakistan,Sri_Lanka,India (accessed May 01, 2021).

[B75] Ng D. H. Ho H. J. Chow A. Wong J. Kyaw W. M. Tan A. (2018). Correlation of clinical illness with viremia in Zika virus disease during an outbreak in Singapore. *BMC Infect. Dis.* 18:301. 10.1186/s12879-018-3211-9 29973158 PMC6030762

[B76] Nimmannitya S. Halstead S. B. Cohen S. N. Margiotta M. R. (1969). Dengue and chikungunya virus infection in man in Thailand, 1962–1964. I. Observations on hospitalized patients with hemorrhagic fever. *Am. J. Trop. Med. Hyg.* 18 954–971. 10.4269/ajtmh.1969.18.954 5355242

[B77] Obeyesekere I. Hermon Y. (1972). Myocarditis and cardiomyopathy after arbovirus infections (dengue and chikungunya fever). *Br. Heart. J.* 34 821–827. 10.1136/hrt.34.8.821 4262698 PMC486987

[B78] Obeyesekere I. Hermon Y. (1973). Arbovirus heart disease: myocarditis and cardiomyopathy following dengue and chikungunya fever – a follow-up study. *Am. Heart. J.* 85 186–194. 10.1016/0002-8703(73)90459-6 4688831

[B79] Onyango M. G. Ciota A. T. Kramer L. D. (2020). The Vector-Host-Pathogen Interface: The Next Frontier in the Battle Against Mosquito-Borne Viral Diseases? *Front. Cell Infect. Microbiol.* 10:547. 10.3389/fcimb.2020.564518 33178624 PMC7596266

[B80] Padbidri V. S. Gnaneswar T. T. (1979). Epidemiological investigations of chikungunya epidemic at Barsi, Maharashtra state, India. *J. Hyg. Epidemiol. Microbiol. Immunol.* 23 445–451. 575900

[B81] Pan American and Health Organization (PAHO) (2021). *Chikungunya: Epidemiological alerts and updates.* Available online at: https://www.paho.org/hq/index.php?option=com_topics&view=rdmore&cid=5855&Itemid=40931&lang=en (accessed Jan 03, 2021).

[B82] Parola P. de Lamballerie X. Jourdan J. Rovery C. Vaillant V. Minodier P. (2006). Novel chikungunya virus variant in travelers returning from Indian Ocean islands. *Emerg. Infect. Dis.* 12 1493–1499. 10.3201/eid1210.060610 17176562 PMC3290960

[B83] Pastorino B. Muyembe-Tamfum J. J. Bessaud M. Tock F. Tolou H. Durand J. P. (2004). Epidemic resurgence of Chikungunya virus in democratic Republic of the Congo: identification of a new central African strain. *J. Med. Virol.* 74 277–282. 10.1002/jmv.20168 15332277

[B84] Paul S. D. Singh K. R. (1968). Experimental infection of *Macaca radiata* with Chikungunya virus and transmission of virus by mosquitoes. *Indian J. Med. Res.* 56 802–811. 4971384

[B85] Pavri K. M. (1964). Presence of chikungunya antibodies in human sera collected from Calcutta and Jamshedpur before 1963. *Indian J. Med. Res.* 52 698–702. 14195510

[B86] Peyrefitte C. N. Rousset D. Pastorino B. A. Pouillot R. Bessaud M. Tock F. (2007). Chikungunya virus, Cameroon, 2006. *Emerg. Infect. Dis.* 13 768–771. 10.3201/eid1305.061500 17553262 PMC2738435

[B87] Phadungsombat J. Imad H. Rahman M. Nakayama E. E. Kludkleeb S. Ponam T. (2020). A Novel Sub-Lineage of Chikungunya Virus East/Central/South African Genotype Indian Ocean Lineage Caused Sequential Outbreaks in Bangladesh and Thailand. *Viruses* 12:1319. 10.3390/v12111319 33213040 PMC7698486

[B88] Pialoux G. Gaüzère B. A. Jauréguiberry S. Strobel M. (2007). Chikungunya, an epidemic arbovirosis. *Lancet Infect. Dis.* 7 319–327. 10.1016/s1473-3099(07)70107-x 17448935

[B89] Porter K. R. Tan R. Istary Y. Suharyono W. Sutaryo, Widjaja S. (2004). A serological study of Chikungunya virus transmission in Yogyakarta, Indonesia: evidence for the first outbreak since 1982. *Southeast Asian J. Trop. Med. Public Health.* 35 408–415. 15691147

[B90] Powers A. M. Brault A. C. Tesh R. B. Weaver S. C. (2000). Reemergence of Chikungunya and O’nyong-nyong viruses: evidence for distinct geographical lineages and distant evolutionary relationships. *J. Gen. Virol.* 81 471–479. 10.1099/0022-1317-81-2-471 10644846

[B91] Powers A. M. Logue C. H. (2007). Changing patterns of chikungunya virus: re-emergence of a zoonotic arbovirus. *J. Gen. Virol.* 88 2363–2377. 10.1099/vir.0.82858-0 17698645

[B92] Presti A. L. Cella E. Angeletti S. Ciccozzi M. (2016). Molecular epidemiology, evolution and phylogeny of Chikungunya virus: an updating review. *Infect. Genet. Evol.* 41 270–278. 10.1016/j.meegid.2016.04.006 27085290

[B93] Pyke A. T. Moore P. R. McMahon J. (2018). New insights into chikungunya virus emergence and spread from Southeast Asia. *Emerg. Microbes. infect* 7:26. 10.1038/s41426-018-0024-2 29535302 PMC5849737

[B94] Rajapakse S. Rodrigo C. Rajapakse A. (2010). Atypical manifestations of chikungunya infection. *Trans. R. Soc. Trop. Med. Hyg.* 104 89–96. 10.1016/j.trstmh.2009.07.031 19716149

[B95] Ravi V. (2006). Re-emergence of chikungunya virus in India. *Indian J. Med. Microbiol.* 24 83–84. 10.1016/s0255-0857(21)02403-8 16687855

[B96] Reiter P. Fontenille D. Paupy C. (2006). Aedes albopictus as an epidemic vector of chikungunya virus: another emerging problem? *Lancet Infect. Dis.* 6 463–464. 10.1016/s1473-3099(06)70531-x 16870524

[B97] Reller M. E. Akoroda U. Nagahawatte A. Devasiri V. Kodikaarachchi W. Strouse J. J. (2013). Chikungunya as a cause of acute febrile illness in southern Sri Lanka. *PLoS One.* 8:e82259. 10.1371/journal.pone.0082259 24312651 PMC3846738

[B98] Renault P. Solet J. L. Sissoko D. Balleydier E. Larrieu S. Filleul L. (2007). A major epidemic of chikungunya virus infection on Reunion Island, France, 2005–2006. *Am. J. Trop. Med. Hyg.* 77 727–731. 10.4269/ajtmh.2007.77.727 17978079

[B99] Riswari S. F. Ma’roef C. N. Djauhari H. Kosasih H. Perkasa A. Yudhaputri F. A. (2016). Study of viremic profile in febrile specimens of chikungunya in Bandung, Indonesia. *J. Clin. Virol.* 74 61–65. 10.1016/j.jcv.2015.11.017 26679829

[B100] Robillard P. Y. Boumahni B. Gerardin P. Michault A. Fourmaintraux A. Schuffenecker I. (2006). Vertical maternal fetal transmission of the chikungunya virus. Ten cases among 84 pregnant women. *Presse Med.* 35 785–788. 10.1016/s0755-4982(06)74690-5 16710146

[B101] Robinson M. C. (1955). An epidemic of virus disease in Southern Province, Tanganyika Territory, in 1952–53. I. Clinical features. *Trans. R. Soc. Trop. Med. Hyg.* 49 28–32. 10.1016/0035-9203(55)90080-8 14373834

[B102] Rodrigues Faria N. Lourenço J. Marques, de Cerqueira E. Maia, de Lima M. (2016). Epidemiology of Chikungunya Virus in Bahia, Brazil, 2014-2015. *PLoS Curr.* 8:ecurrents.outbreaks.c97507e3e48efb946401755d468c28b2. 10.1371/currents.outbreaks.c97507e3e48efb946401755d468c28b2 27330849 PMC4747681

[B103] Rodriguez-Morales A. J. Villamil-Gómez W. E. Franco-Paredes C. (2016). The arboviral burden of disease caused by co-circulation and co-infection of dengue, chikungunya and Zika in the Americas. *Travel. Med. Infect. Dis.* 4 177–179. 10.1016/j.tmaid.2016.05.004 27224471

[B104] Ross R. W. (1956). The Newala epidemic. III. The virus: isolation, pathogenic properties and relationship to the epidemic. *J. Hyg.* 54 177–191. 10.1017/s0022172400044442 13346078 PMC2218030

[B105] Saxena S. K. Singh M. Mishra N. Lakshmi V. (2006). Resurgence of chikungunya virus in India: an emerging threat. *Euro. Surveill.* 11 E060810–E060812. 10.2807/esw.11.32.03019-en 16966777

[B106] Schlesinger M. Schlesinger S. (1986). “Formation and assembly of alphavirus glycoproteins,” in *The Togaviridae and Flaviviridae*, eds Schlesinger S. Schlesinger M. J. (New York: Plenum Publishing Corp), 121–148. 10.1007/978-1-4757-0785-4_5

[B107] Schuffenecker I. Iteman I. Michault A. Murri S. Frangeul L. Vaney M. C. (2006). Genome microevolution of chikungunya viruses causing the Indian Ocean outbreak. *PLoS Med.* 3:e263. 10.1371/journal.pmed.0030263 16700631 PMC1463904

[B108] Scolari F. Casiraghi M. Bonizzoni M. (2019). Aedes spp. and their microbiota: a review. *Front. Microbial.* 10:2036. 10.3389/fmicb.2019.02036 31551973 PMC6738348

[B109] Sergon K. Yahaya A. A. Brown J. Bedja S. A. Agata N. Allaranger Y. (2007). Seroprevalence of chikungunya virus infection on Grande Comore Island, Union of the Comoros, March 2005. *Am. J. Trop. Med. Hyg.* 76 1189–1193. 10.4269/ajtmh.2007.76.1189 17556634

[B110] Seyler T. Hutin Y. Ramanchandran V. Ramakrishnan R. Manickam P. Murhekar M. (2010). Estimating the burden of disease and the economic cost attributable to chikungunya, Andhra Pradesh, India, 2005–2006. *Trans. R. Soc. Trop. Med. Hyg.* 104 133–138. 10.1016/j.trstmh.2009.07.014 19709705

[B111] Shen J. Y. Li M. Xie L. Mao J. R. Zhou H. N. Wang P. G. (2020). Perinatal Vertical Transmission of Chikungunya Virus in Ruili, a Town on the Border between China and Burma. *Virol. Sin* 36 145–148. 10.1007/s12250-020-00245-y 32677020 PMC7364398

[B112] Silva L. A. Dermody T. S. (2017). Chikungunya virus: epidemiology, replication, disease mechanisms, and prospective intervention strategies. *J. Clin. Investig.* 127 737–749. 10.1172/JCI84417 28248203 PMC5330729

[B113] Silva J. V. Jr. Ludwig-Begall L. F. de Oliveira-Filho E. F. Oliveira R. A. Durães-Carvalho R. Lopes T. R. (2018). A scoping review of Chikungunya virus infection: epidemiology, clinical characteristics, viral co-circulation complications, and control. *Acta. Trop.* 188 213–224. 10.1016/j.actatropica.2018.09.003 30195666 PMC7092809

[B114] Simizu B. Yamamoto K. Hashimoto K. Ogata T. (1984). Structural proteins of Chikungunya virus. *J. Virol.* 51 254–258. 10.1128/jvi.51.1.254-258.1984 6726893 PMC254427

[B115] Simo F. B. N. Bigna J. J. Well E. A. Kenmoe S. Sado F. B. Y. Weaver S. C. (2019). Chikungunya virus infection prevalence in Africa: a contemporaneous systematic review and meta-analysis. *Public Health.* 166 79–88. 10.1016/j.puhe.2018.09.027 30468973

[B116] Soekiman S. (1987). A study on susceptibility of Indonesia colonies of Aedes aegypti and Aedes albopictus mosquitoes to experimental infection with dengue type 3 and chikungunya viruses. *Kobe J. Med. Sci.* 33 19–34. 3586577

[B117] Soekiman S. Matsumura T. Yamanishi H. (1986b). Multiplication of chikungunya virus in salivary glands of Aedes albopictus (Oahu strain) mosquitoes: an electron microscopic study. *JPN. J. Med. Sci. Biol.* 39 207–211. 10.7883/yoken1952.39.207 3599528

[B118] Soekiman S. Konishi E. J. Matsumura T. (1986a). Susceptibility of Indonesia colonies of Aedes aegypti and Aedes albopictus mosquitoes to experimental infection with chikungunya virus. *Kobe J. Med. Sci.* 32 127–132. 3023740

[B119] Spence L. P. Thomas L. (1959). Application of haemagglutination and complement fixation techniques to the identification and serological classification of arthropod-borne viruses; studies on Chikungunya and Makonde viruses. *Trans. R. Soc. Trop. Med. Hyg.* 53 248–255. 10.1016/0035-9203(59)90004-5 13669029

[B120] Spicher T. Delitz M. Schneider A. D. B. Wolfinger M. T. (2021). Dynamic molecular epidemiology reveals lineage-associated single-nucleotide variants that alter RNA structure in Chikungunya virus. *Genes* 12:239. 10.3390/genes12020239 33567556 PMC7914560

[B121] Staples J. E. Breiman R. F. Powers A. M. (2009). Chikungunya fever: an epidemiological review of a re-emerging infectious disease. *Clin. Infect. Dis.* 49 942–948. 10.1086/605496 19663604

[B122] Suhrbier A. (2019). Rheumatic manifestations of chikungunya: emerging concepts and interventions. *Nat. Rev. Rheumatol.* 15 597–611. 10.1038/s41584-019-0276-9 31481759

[B123] Tesh R. B. (1982). Arthritides caused by mosquito-borne viruses. *Annu. Rev. Med.* 33 31–40. 10.1146/annurev.me.33.020182.000335 6123291

[B124] Thaung U. Ming C. K. Swe T. Thein S. (1975). Epidemiological features of dengue and chikungunya infections in Burma. *Southeast Asian J. Trop. Med. Public Health.* 6 276–283. 126493

[B125] Thein S. La Linn M. Aaskov J. Aung M. M. Aye M. Zaw A. (1992). Development of a simple indirect enzyme-linked immunosorbent assay for the detection of immunoglobulin M antibody in serum from patients following an outbreak of chikungunya virus infection in Yangon, Myanmar. *Trans. R. Soc. Trop. Med. Hyg.* 86 438–442. 10.1016/0035-9203(92)90260-J 1332222

[B126] Thonnon J. Spiegel A. Diallo M. Diallo A. Fontenille D. (1999). Chikungunya virus outbreak in Senegal in 1996 and 1997. *Bull. Soc. Pathol. Exot.* 92 79–82. 10399593

[B127] Tsetsarkin K. A. Chen R. Weaver S. C. (2016). Interspecies transmission and chikungunya virus emergence. *Curr. Opin. Virol.* 16 143–150. 10.1016/j.coviro.2016.02.007 26986235 PMC4824623

[B128] Vairo F. Haider N. Kock R. Ntoumi F. Ippolito G. Zumla A. (2019). Chikungunya: epidemiology, pathogenesis, clinical features, management, and prevention. *Infect. Dis. Clin. North. Am.* 33 1003–1025. 10.1016/j.idc.2019.08.006 31668189

[B129] van Enter B. J. Huibers M. H. van Rooij L. Steingrover R. van Hensbroek M. B. Voigt R. R. (2018). Perinatal outcomes in vertically infected neonates during a chikungunya outbreak on the island of Curacao. *Am. J. Trop. Med. Hyg.* 99 1415–1418. 10.4269/ajtmh.17-0957 30328407 PMC6283481

[B130] Vazeille M. Moutailler S. Coudrier D. Rousseaux C. Khun H. Huerre M. (2007). Two Chikungunya isolates from the outbreak of La Reunion (Indian Ocean) exhibit different patterns of infection in the mosquito. *Aedes albopictus. PLoS One.* 2:e1168. 10.1371/journal.pone.0001168 18000540 PMC2064959

[B131] Villamil-Gómez W. E. Rodríguez-Morales A. J. Uribe-García A. M. González-Arismendy E. Castellanos J. E. Calvo E. P. (2016). Zika, dengue, and chikungunya co-infection in a pregnant woman from Colombia. *Int. J. Infect. Dis.* 51 135–138. 10.1016/j.ijid.2016.07.017 27497951

[B132] Wahid B. Ali A. Rafique S. Idrees M. (2017). Global expansion of chikungunya virus: mapping the 64-year history. *Int. J. Infect. Dis.* 58 69–76. 10.1016/j.ijid.2017.03.006 28288924

[B133] Weaver S. C. Forrester N. L. (2015). Chikungunya: Evolutionary history and recent epidemic spread. *Antivir. Res.* 120 32–39. 10.1016/j.antiviral.2015.04.016 25979669

[B134] Weaver S. C. Frey T. K. Huang H. V. Kinney R. M. Rice C. M. Roehrig J. T. (2005). “Togaviridae,” in *Virus Taxonomy: Eighth Report of the International Committee on Taxonomy of Viruses*, Fauquet C. Mayo M. A. Maniloff J. Desselberger U. Ball L. A. (Amsterda: Elsevier), 999–1008.

[B135] Weaver S. C. Lecuit M. (2015). Chikungunya virus and the global spread of a mosquito-borne disease. *N. Engl. J. Med.* 372 1231–1239. 10.1056/NEJMra1406035 25806915

[B136] Wimalasiri-Yapa B. R. Stassen L. Huang X. Hafner L. M. Hu W. Devine G. J. (2019). Chikungunya virus in Asia–Pacific: a systematic review. *Emerg. Microbes. infect.* 8 70–79. 10.1080/22221751.2018.1559708 30866761 PMC6455125

[B137] World Health and Organization (WHO) (2021). *Chikungunya.* Available online at: https://www.who.int/news-room/fact-sheets/detail/chikungunya (accessed Jan 03, 2021).

[B138] Yactayo S. Staples J. E. Millot V. Cibrelus L. Ramon-Pardo P. (2016). Epidemiology of Chikungunya in the Americas. *J. Infect. Dis.* 214 S441–S445. 10.1093/infdis/jiw390 27920170 PMC5137246

[B139] Yadav P. Shouche Y. S. Munot H. P. Mishra A. C. Mourya D. T. (2003). Genotyping of Chikungunya virus isolates from India during 1963–2000 by reverse transcription-polymerase chain reaction. *Acta. Virol.* 47 125–127. 14524480

[B140] Zanotto P. M. D. A. Leite L. C. D. C. (2018). The challenges imposed by Dengue, Zika, and Chikungunya to Brazil. *Front. Immunol.* 9:1964. 10.3389/fimmu.2018.01964 30210503 PMC6121005

[B141] Zim M. M. Sam I. C. Omar S. S. Chan Y. F. AbuBakar S. Kamarulzaman A. (2013). Chikungunya infection in Malaysia: comparison with dengue infection in adults and predictors of persistent arthralgia. *J. Clin. Virol.* 56 141–145. 10.1016/j.jcv.2012.10.019 23201456

