# Supplementary material for: Molecular Epidemiology, Evolution and Reemergence of Chikungunya Virus in South Asia
Source: Front Microbiol. 2021 Jun 7;12:689979. doi: 10.3389/fmicb.2021.689979 (PMC8215147; doi:10.3389/fmicb.2021.689979)
Supplement: Supplementary Table 1 — Documented local outbreaks of chikungunya virus with the distribution of vector species during 1950s to 2020 from more than 110 countries and territories worldwide. Data were retrieved from Centers for Disease Control and Prevention (https://www.cdc.gov/chikungunya/index.html), World Health Organization (https://www.who.int/news-room/fact-sheets/detail/chikungunya), European Centre for Disease Prevention and Control (https://www.ecdc.europa.eu/en/chikungunya-monthly), and Pan American Health Organization (https://www.paho.org/hq/index.php?option=com_topics&view= rdmore&cid=5855&Itemid=40931&lang=en). [file Table_1.docx]

**Supplementary Table 1.** Documented local outbreaks of chikungunya virus with the distribution of vector species during 1950s to 2020 from more than 101 countries and territories worldwide. Data were retrieved from Centers for Disease Control and Prevention (<https://www.cdc.gov/chikungunya/index.html>), World Health Organization (<https://www.who.int/news-room/fact-sheets/detail/chikungunya>), European Centre for Disease Prevention and Control (<https://www.ecdc.europa.eu/en/chikungunya-monthly>) and Pan American Health Organization (<https://www.paho.org/hq/index.php?option=com_topics&view=rdmore&cid=5855&Itemid=40931&lang=en>).

| **Continents** | **Countries and territories** | **Year of outbreaks** | **Genotypes** | **Vectors** | **References** |
| --- | --- | --- | --- | --- | --- |
| Asia | ***Bangladesh*** | 2008, 2011, 2013, 2017 | Asian, ECSA | *Aedes aegypti, Aedes albopictus* | (CDC, 2021; Wahid et al., 2017; Wimalasiri-Yapa et al., 2019) |
|  | ***Bhutan*** | 2012-2016, 2019 | Asian, ECSA, IOL | *Aedes aegypti, Aedes albopictus* | (CDC, 2021; Wahid et al., 2017; Wimalasiri-Yapa et al., 2019) |
|  | Cambodia | 1961, 1963, 2008-2012, 2020 | Asian, ECSA, IOL | *Aedes aegypti, Aedes albopictus* | (CDC, 2021; Wahid et al., 2017; Wimalasiri-Yapa et al., 2019) |
|  | China | 1987, 2008-2010, 2017 | Asian, ECSA | *Aedes aegypti, Aedes albopictus* | (CDC, 2021; Wahid et al., 2017; Wimalasiri-Yapa et al., 2019) |
|  | ***India*** | 1963–1965, 1973, 2006-2011, 2014, 2016-2018 | Asian, ECSA, IOL | *Aedes aegypti, Aedes albopictus* | (CDC, 2021; Wahid et al., 2017; Wimalasiri-Yapa et al., 2019) |
|  | Indonesia | 1973, 1980, 1983–1984, 1998–1999, 2000–2005, 2009-2019 | Asian, ECSA | *Aedes aegypti, Aedes albopictus* | (CDC, 2021; Wahid et al., 2017; Wimalasiri-Yapa et al., 2019) |
|  | Laos | 2005, 2017, 2019 | ECSA | *Aedes aegypti, Aedes albopictus* | (CDC, 2021; Wahid et al., 2017; Wimalasiri-Yapa et al., 2019) |
|  | Malaysia | 1965–1969, 1998–1999, 2006, 2008, 2009-2019 | Asian, ECSA, IOL | *Aedes aegypti, Aedes albopictus* | (CDC, 2021; Wahid et al., 2017; Wimalasiri-Yapa et al., 2019) |
|  | ***Maldives*** | 2006, 2019, 2020 | Asian, ECSA | *Aedes aegypti, Aedes albopictus* | (CDC, 2021; Wahid et al., 2017; Wimalasiri-Yapa et al., 2019) |
|  | Myanmar | 1973, 2004, 2010, 2015, 2019 | Asian, ECSA | *Aedes aegypti, Aedes albopictus* | (CDC, 2021; Wahid et al., 2017; Wimalasiri-Yapa et al., 2019) |
|  | ***Nepal*** | 2014, 2015, 2017 | Asian, ECSA | *Aedes aegypti, Aedes albopictus* | (CDC, 2021; Wahid et al., 2017; Wimalasiri-Yapa et al., 2019) |
|  | ***Pakistan*** | 1983, 2016, 2017 | Asian, ECSA | *Aedes albopictus* | (CDC, 2021; Wahid et al., 2017; Wimalasiri-Yapa et al., 2019) |
|  | Philippines | 1985–1986, 2011, 2012 | Asian, ECSA | *Aedes aegypti, Aedes albopictus* | (CDC, 2021; Wahid et al., 2017; Wimalasiri-Yapa et al., 2019) |
|  | Saudi Arabia | 1997, 2011 | Asian | *Aedes albopictus* | (CDC, 2021; Wahid et al., 2017; Wimalasiri-Yapa et al., 2019) |
|  | Singapore | 2007-2009 | Asian, ECSA, IOL | *Aedes aegypti, Aedes albopictus* | (CDC, 2021; Wahid et al., 2017; Wimalasiri-Yapa et al., 2019) |
|  | ***Sri Lanka*** | 1965, 2006-2009 | Asian, ECSA, IOL | *Aedes aegypti, Aedes albopictus* | (CDC, 2021; Wahid et al., 2017; Wimalasiri-Yapa et al., 2019) |
|  | Taiwan | 1967, 2006, 2019 | Asian, ECSA | *Aedes albopictus* | (CDC, 2021; Wahid et al., 2017; Wimalasiri-Yapa et al., 2019) |
|  | Thailand | 1960, 1962–1964, 1988, 1991–1993, 1995, 2008-2010, 2014-2016, 2019 | Asian, ECSA | *Aedes aegypti, Aedes albopictus* | (CDC, 2021; Wahid et al., 2017; Wimalasiri-Yapa et al., 2019) |
|  | Timor-Leste | 2007-2010 | Asian, ECSA | *Aedes aegypti, Aedes albopictus* | (CDC, 2021; Wahid et al., 2017; Wimalasiri-Yapa et al., 2019) |
| Asia | Vietnam | 1965, 1967, 2015-2019 | Asian, ECSA | *Aedes aegypti, Aedes albopictus* | (CDC, 2021; Wahid et al., 2017; Wimalasiri-Yapa et al., 2019) |
|  | Yemen | 2011, 2019 | Asian, ECSA | *Aedes aegypti, Aedes albopictus* | (CDC, 2021; Wahid et al., 2017; Wimalasiri-Yapa et al., 2019) |
| Africa | Angola | 1970, 1971, 2004 | ECSA | *Aedes aegypti* | (Powers & Logue, 2007; Wahid et al., 2017; Weaver & Forrester, 2015) |
|  | Benin | 2006, 2007, 2014, 2015 | ECSA | *Aedes aegypti* | (Powers & Logue, 2007; Wahid et al., 2017; Weaver & Forrester, 2015) |
|  | Burundi | 1980–1982, 2014 | ECSA | *Aedes aegypti, Aedes albopictus* | (Powers & Logue, 2007; Wahid et al., 2017; Weaver & Forrester, 2015) |
|  | Cameroon | 2004, 2006, 2009-2014 | ECSA | *Aedes aegypti, Aedes albopictus* | (Powers & Logue, 2007; Wahid et al., 2017; Weaver & Forrester, 2015) |
|  | CAR | 1978, 1979, 1999–2000, 2014, 2016 | ECSA | *Aedes aegypti, Aedes albopictus* | (Powers & Logue, 2007; Wahid et al., 2017; Weaver & Forrester, 2015) |
|  | Chad | 2004-2005, 2014, 2020 | ECSA | *Aedes aegypti, Aedes albopictus* | (Powers & Logue, 2007; Wahid et al., 2017; Weaver & Forrester, 2015) |
|  | Cote d’Ivoire | 2011, 2013, 2015 | ECSA | *Aedes aegypti, Aedes albopictus* | (Powers & Logue, 2007; Wahid et al., 2017; Weaver & Forrester, 2015) |
|  | Djibouti | 2005-2007, 2011 | ECSA | *Aedes aegypti* | (Powers & Logue, 2007; Wahid et al., 2017; Weaver & Forrester, 2015) |
|  | DRC | 2004-2007, 2009-2012 | ECSA | *Aedes aegypti, Aedes albopictus* | (Powers & Logue, 2007; Wahid et al., 2017; Weaver & Forrester, 2015) |
|  | Equatorial Guinea | 2002, 2004-2006 | ECSA | *Aedes aegypti, Aedes albopictus* | (Powers & Logue, 2007; Wahid et al., 2017; Weaver & Forrester, 2015) |
|  | Ethiopia | 2004-2006, 2013, 2020 | ECSA | *Aedes aegypti* | (Powers & Logue, 2007; Wahid et al., 2017; Weaver & Forrester, 2015) |
|  | Gabon | 1982, 2007-2008, 2010, 2011 | ECSA | *Aedes aegypti, Aedes albopictus* | (Powers & Logue, 2007; Wahid et al., 2017; Weaver & Forrester, 2015) |
|  | Guinea | 2002, 2006 | West African | *Aedes aegypti* | (Powers & Logue, 2007; Wahid et al., 2017; Weaver & Forrester, 2015) |
|  | Kenya | 1970, 2004, 2005, 2009-2012, 2016 | ECSA, IOL | *Aedes aegypti* | (Powers & Logue, 2007; Wahid et al., 2017; Weaver & Forrester, 2015) |
|  | Madagascar | 2006, 2007-2014 | IOL, ECSA | *Aedes aegypti, Aedes albopictus* | (Powers & Logue, 2007; Wahid et al., 2017; Weaver & Forrester, 2015) |
|  | Malawi | 1987–1989 | ECSA | *Aedes aegypti, Aedes albopictus* | (Powers & Logue, 2007; Wahid et al., 2017; Weaver & Forrester, 2015) |
|  | Mauritius | 2005,2006 | IOL, ECSA | *Aedes aegypti* | (Powers & Logue, 2007; Wahid et al., 2017; Weaver & Forrester, 2015) |
|  | Mayotte | 2006 | ECSA | *Aedes aegypti, Aedes albopictus* | (Powers & Logue, 2007; Wahid et al., 2017; Weaver & Forrester, 2015) |
|  | Mozambique | 2015-2017 | ECSA | *Aedes aegypti* | (Powers & Logue, 2007; Wahid et al., 2017; Weaver & Forrester, 2015) |
|  | Nigeria | 1964, 1969, 1974–1975, 1980, 2004-2007, 2017 | West African | *Aedes aegypti, Aedes albopictus* | (Powers & Logue, 2007; Wahid et al., 2017; Weaver & Forrester, 2015) |
|  | Republic of the Congo | 1958, 1960,1999, 2000, 2011 | ECSA, IOL | *Aedes aegypti* | (Powers & Logue, 2007; Wahid et al., 2017; Weaver & Forrester, 2015) |
|  | Reunion | 2004-2006, 2001-2012 | ECSA | *Aedes aegypti* | (Powers & Logue, 2007; Wahid et al., 2017; Weaver & Forrester, 2015) |
|  | Senegal | 1966, 1982, 1996–1997,2015 | West African, ECSA | *Aedes aegypti* | (Powers & Logue, 2007; Wahid et al., 2017; Weaver & Forrester, 2015) |
|  | Seychelles | 2005, 2006 | IOL, ECSA | *Aedes aegypti, Aedes albopictus* | (Powers & Logue, 2007; Wahid et al., 2017; Weaver & Forrester, 2015) |
|  | Sierra Leone | 1972, 2013-2015 | ECSA | *Aedes aegypti* | (Powers & Logue, 2007; Wahid et al., 2017; Weaver & Forrester, 2015) |
|  | Somalia | 1960, 1996-97, 2014, 2015, 2016 | ECSA | *Aedes aegypti* | (Powers & Logue, 2007; Wahid et al., 2017; Weaver & Forrester, 2015) |
|  | South Africa | 1956, 1970, 1975-77, 2004-2007, 2014-2017 | ECSA | *Aedes aegypti* | (Powers & Logue, 2007; Wahid et al., 2017; Weaver & Forrester, 2015) |
|  | Sudan | 2005, 2007, 2018 | ECSA | *Aedes aegypti* | (Powers & Logue, 2007; Wahid et al., 2017; Weaver & Forrester, 2015) |
|  | Tanzania | 1952, 2007-2008, 2014 | ECSA | *Aedes aegypti* | (Powers & Logue, 2007; Wahid et al., 2017; Weaver & Forrester, 2015) |
|  | Uganda | 1958, 1961, 1962, 1971, 1985, 2004, 2007, 2015 | ECSA | *Aedes aegypti* | (Powers & Logue, 2007; Wahid et al., 2017; Weaver & Forrester, 2015) |
|  | Zimbabwe | 1957, 1961-1963,1971, 2004-2007, 2013, 2014 | ECSA | *Aedes aegypti* | (Powers & Logue, 2007; Wahid et al., 2017; Weaver & Forrester, 2015) |
| America | Anguilla | 2016 | Asian, ECSA | *Aedes albopictus* | (CDC, 2021; PHAO, 2021; WHO, 2021) |
|  | Antigua and Barbuda | 2016 | Asian, ECSA | *Aedes albopictus* | (CDC, 2021; PHAO, 2021; WHO, 2021) |
|  | Argentina | 2016 | Asian, ECSA | *Aedes albopictus* | (CDC, 2021; PHAO, 2021; WHO, 2021) |
|  | Aruba | 2015 | Asian, ECSA | *Aedes aegypti, Aedes albopictus* | (CDC, 2021; PHAO, 2021; WHO, 2021) |
|  | Bahamas | 2014 | Asian, ECSA | *Aedes aegypti, Aedes albopictus* | (CDC, 2021; PHAO, 2021; WHO, 2021) |
|  | Barbados | 2014, 2016 | Asian, ECSA | *Aedes albopictus* | (CDC, 2021; PHAO, 2021; WHO, 2021) |
|  | Belize | 2016 | Asian, ECSA | *Aedes albopictus* | (CDC, 2021; PHAO, 2021; WHO, 2021) |
|  | Bolivia | 2016, 2018-2020 | Asian, ECSA | *Aedes albopictus* | (CDC, 2021; PHAO, 2021; WHO, 2021) |
|  | Brazil | 2016, 2018-2020 | Asian, ECSA | *Aedes aegypti, Aedes albopictus* | (CDC, 2021; PHAO, 2021; WHO, 2021) |
|  | Colombia | 2014, 2015, 2018-2020 | Asian, ECSA | *Aedes aegypti, Aedes albopictus* | (CDC, 2021; PHAO, 2021; WHO, 2021) |
|  | Costa Rica | 2015 | Asian, ECSA | *Aedes aegypti, Aedes albopictus* | (CDC, 2021; PHAO, 2021; WHO, 2021) |
|  | Cuba | 2015 | Asian, ECSA | *Aedes aegypti, Aedes albopictus* | (CDC, 2021; PHAO, 2021; WHO, 2021) |
|  | Dominica | 2014 | Asian, ECSA | *Aedes aegypti, Aedes albopictus* | (CDC, 2021; PHAO, 2021; WHO, 2021) |
|  | Dominican Republic | 2014 | Asian, ECSA | *Aedes albopictus* | (CDC, 2021; PHAO, 2021; WHO, 2021) |
|  | Ecuador | 2015 | Asian, ECSA | *Aedes albopictus* | (CDC, 2021; PHAO, 2021; WHO, 2021) |
|  | El Salvador | 2015 | Asian, ECSA | *Aedes albopictus* | (CDC, 2021; PHAO, 2021; WHO, 2021) |
|  | Grenada | 2015 | Asian, ECSA | *Aedes albopictus* | (CDC, 2021; PHAO, 2021; WHO, 2021) |
|  | Guadeloupe | 2014 | Asian, ECSA | *Aedes albopictus* | (CDC, 2021; PHAO, 2021; WHO, 2021) |
|  | Guatemala | 2015 | Asian, ECSA | *Aedes albopictus* | (CDC, 2021; PHAO, 2021; WHO, 2021) |
|  | Guyana | 1992, 2014 | Asian, ECSA | *Aedes albopictus* | (CDC, 2021; PHAO, 2021; WHO, 2021) |
|  | Haiti | 2014 | Asian, ECSA | *Aedes albopictus* | (CDC, 2021; PHAO, 2021; WHO, 2021) |
|  | Honduras | 2015 | Asian, ECSA | *Aedes albopictus* | (CDC, 2021; PHAO, 2021; WHO, 2021) |
|  | Jamaica | 2014 | Asian, ECSA | *Aedes albopictus* | (CDC, 2021; PHAO, 2021; WHO, 2021) |
|  | Mexico | 2014, 2015, 2018-2020 | Asian, ECSA | *Aedes albopictus* | (CDC, 2021; PHAO, 2021; WHO, 2021) |
|  | Nicaragua | 2016 | Asian, ECSA | *Aedes albopictus* | (CDC, 2021; PHAO, 2021; WHO, 2021) |
|  | Panama | 2017 | Asian, ECSA | *Aedes albopictus* | (CDC, 2021; PHAO, 2021; WHO, 2021) |
|  | Paraguay | 2015 | Asian, ECSA | *Aedes albopictus* | (CDC, 2021; PHAO, 2021; WHO, 2021) |
|  | Peru | 2014, 2018-2020 | Asian, ECSA | *Aedes albopictus* | (CDC, 2021; PHAO, 2021; WHO, 2021) |
|  | Saint Barthelemy | 2012, 2013, 2017 | Asian, ECSA | *Aedes albopictus* | (CDC, 2021; PHAO, 2021; WHO, 2021) |
|  | Saint Kitts and Nevis | 2013, 2014 | Asian, ECSA | *Aedes albopictus* | (CDC, 2021; PHAO, 2021; WHO, 2021) |
|  | Saint Lucia | 2013, 2014 | Asian, ECSA | *Aedes albopictus* | (CDC, 2021; PHAO, 2021; WHO, 2021) |
|  | Suriname | 2014 | Asian, ECSA | *Aedes albopictus* | (CDC, 2021; PHAO, 2021; WHO, 2021) |
|  | Trinidad and Tobago | 2015 | Asian, ECSA | *Aedes albopictus* | (CDC, 2021; PHAO, 2021; WHO, 2021) |
|  | United States | 1985–1986,2006, 2014, 2018 | Asian, ECSA | *Aedes aegypti, Aedes albopictus* | (CDC, 2021; PHAO, 2021; WHO, 2021) |
| Americas | Venezuela | 2015, 2018-2020 | Asian, ECSA | *Aedes aegypti, Aedes albopictus* | (CDC, 2021; PHAO, 2021; WHO, 2021) |
| Oceania | American Samoa | 2014 | ECSA | *Aedes albopictus* | (CDC, 2021; WHO, 2021) |
|  | Cook Islands | 2011-2014 | Not specified | *Aedes albopictus* | (CDC, 2021; WHO, 2021) |
|  | Federal States of Micronesia | 2011-2014 | ECSA | *Aedes albopictus* | (CDC, 2021; WHO, 2021) |
|  | Fiji | 2011-2014 | ECSA | *Aedes albopictus* | (CDC, 2021; WHO, 2021) |
|  | French Polynesia | 2014 | ECSA | *Aedes aegypti, Aedes albopictus* | (CDC, 2021; WHO, 2021) |
|  | Kiribati | 2011-2014 | ECSA | *Aedes albopictus* | (CDC, 2021; WHO, 2021) |
|  | Marshall Islands | 2011-2014 | ECSA | *Aedes albopictus* | (CDC, 2021; WHO, 2021) |
|  | New Caledonia | 2011-2014 | Not specified | *Aedes albopictus* | (CDC, 2021; PHAO, 2021; WHO, 2021) |
|  | Papua New Guinea | 2012 | ECSA | *Aedes albopictus* | (CDC, 2021; PHAO, 2021; WHO, 2021) |
|  | Samoa | 2014 | Not specified | *Aedes albopictus* | (CDC, 2021; PHAO, 2021; WHO, 2021) |
|  | Tokelau | 2011-2014 | ECSA | *Aedes aegypti, Aedes albopictus* | (CDC, 2021; PHAO, 2021; WHO, 2021) |
|  | Tonga | 2011-2014 | ECSA | *Aedes albopictus* | (CDC, 2021; PHAO, 2021; WHO, 2021) |
| Europe | France | 2010, 2017 | ECSA, IOL | *Aedes albopictus* | (CDC, 2021; ECDC, 2021; WHO, 2021) |
|  | Italy | 2007, 2017 | ECSA, IOL | *Aedes albopictus* | (CDC, 2021; ECDC, 2021; WHO, 2021) |

Italic bold indicates South Asian countries.
